# Unravelling cell type-specific responses to Parkinson’s Disease at single cell resolution

**DOI:** 10.1186/s13024-023-00699-0

**Published:** 2024-01-20

**Authors:** Araks Martirosyan, Rizwan Ansari, Francisco Pestana, Katja Hebestreit, Hayk Gasparyan, Razmik Aleksanyan, Silvia Hnatova, Suresh Poovathingal, Catherine Marneffe, Dietmar R. Thal, Andrew Kottick, Victor J. Hanson-Smith, Sebastian Guelfi, William Plumbly, T. Grant Belgard, Emmanouil Metzakopian, Matthew G. Holt

**Affiliations:** 1 VIB Center for Brain & Disease Research, KU Leuven, Leuven, Belgium; 2 UK Dementia Research Institute, Department of Clinical Neurosciences, University of Cambridge, Cambridge, CB2 0AH UK; 3 Verge Genomics, South San Francisco, CA USA; 4 Armenian Bioinformatics Institute, Yerevan, Armenia; 5 Department of Mathematics and Mechanics, Yerevan State University, Yerevan, Armenia; 6 Laboratory for Neuropathology, Department of Imaging and Pathology and Leuven Brain Institute, KU Leuven, and Department of Pathology, UZ Leuven, Leuven, Belgium; 7 The Bioinformatics CRO, Orlando, FL USA; 8 bit.bio, The Dorothy Hodgkin Building, Babraham Research Institute, Cambridge, CB22 3FH UK; 9 Laboratory of Synapse Biology, i3S, Porto, Portugal

## Abstract

Parkinson’s Disease (PD) is the second most common neurodegenerative disorder. The pathological hallmark of PD is loss of dopaminergic neurons and the presence of aggregated α-synuclein, primarily in the substantia nigra pars compacta (SNpc) of the midbrain. However, the molecular mechanisms that underlie the pathology in different cell types is not currently understood. Here, we present a single nucleus transcriptome analysis of human post-mortem SNpc obtained from 15 sporadic Parkinson’s Disease (PD) cases and 14 Controls. Our dataset comprises ∼84K nuclei, representing all major cell types of the brain, allowing us to obtain a transcriptome-level characterization of these cell types. Importantly, we identify multiple subpopulations for each cell type and describe specific gene sets that provide insights into the differing roles of these subpopulations. Our findings reveal a significant decrease in neuronal cells in PD samples, accompanied by an increase in glial cells and T cells. Subpopulation analyses demonstrate a significant depletion of tyrosine hydroxylase (*TH*) enriched astrocyte, microglia and oligodendrocyte populations in PD samples, as well as *TH* enriched neurons, which are also depleted. Moreover, marker gene analysis of the depleted subpopulations identified 28 overlapping genes, including those associated with dopamine metabolism (e.g., *ALDH1A1, SLC6A3 & SLC18A2*). Overall, our study provides a valuable resource for understanding the molecular mechanisms involved in dopaminergic neuron degeneration and glial responses in PD, highlighting the existence of novel subpopulations and cell type-specific gene sets.

**Supplementary Information:**

The online version contains supplementary material available at 10.1186/s13024-023-00699-0.

## Introduction

Parkinson’s disease (PD) is a progressive neurodegenerative disorder, predominantly affecting the elderly. Clinically, PD is characterized by resting tremors, slowness of movement, rigidity, and postural instability [1]. Degeneration of dopaminergic (DA) neurons and the subsequent loss of the neurotransmitter dopamine within the substantia nigra pars compacta (SNpc) underlies the pathophysiology of the motor dysfunction characteristic of the disease. In the surviving neurons, accumulation of so-called Lewy bodies (protein aggregates composed primarily of alpha-synuclein) is observed and is recognized as a primary (anatomical) hallmark of PD [1, 2].

However, our understanding of the molecular mechanisms underlying PD pathology remains poor. In this respect, with the application of high-throughput single cell/nucleus RNA sequencing (RNA-seq) technologies, we are beginning to tease apart the cell type-specific responses underlying PD [3]. In recent years, RNA-seq has been applied to various human models of PD, using both in vitro systems and ex vivo post-mortem human brain tissues [4–6]. While the latter approach has advantages in fully capturing the complexity of human tissue and its response to disease, obtaining a high-quality post-mortem dataset has proved problematic, due to the scarcity of human brain tissue, and RNA-degradation leading to poor RNA quality. This is especially true for tyrosine hydroxylase (*TH*) positive dopaminergic neurons in PD samples, which are more vulnerable to degeneration. Previous studies by Agarwal et al., 2020 [7] and Smajić et al., 2022 [6], using substantia nigra (SN) samples or midbrain samples respectively, captured less than 200 dopaminergic (DA) neurons. In contrast, Kamath et al., 2022 [8] specifically enriched for dopaminergic neurons by employing a NR4A2 antibody-based enrichment strategy, capturing ~ 22,000 DA neurons from human substantia nigra. While Kamath and colleagues took this approach to focus on the vulnerability of DA neurons in PD, other cell types were also captured (although at much lower levels) and were not included in further analyses.

In this study, we present a unique large-scale single nucleus RNA-seq dataset of 29 post-mortem human brains (14 Controls and 15 PD), specifically targeting SNpc, as it is the region most vulnerable to PD pathology. The dataset includes the transcriptomes of ∼84K high-quality nuclei at > 40% sequencing saturation rate and ∼40K average read depth. Our analyses were able to detect all the major cell types, including a large population of DA neurons (more than 2,000 nuclei) derived from SNpc. In addition to neurons, we also captured large populations of *TH* enriched glial cells. To our knowledge, this aspect of glial heterogeneity in the SNpc has not been discussed in previous studies. We observed a significant depletion of both *TH* enriched neuron and glial populations in PD, and link this to the upregulation of genes associated with the unfolded protein response (UPR) and oxidative stress. Our results point to possible shared molecular mechanisms of neuronal and glial cells that are impacted by PD pathology.

## Results

### Single nucleus RNA-seq reveals cell type heterogeneity in human SNpc

**Fig. 1:**
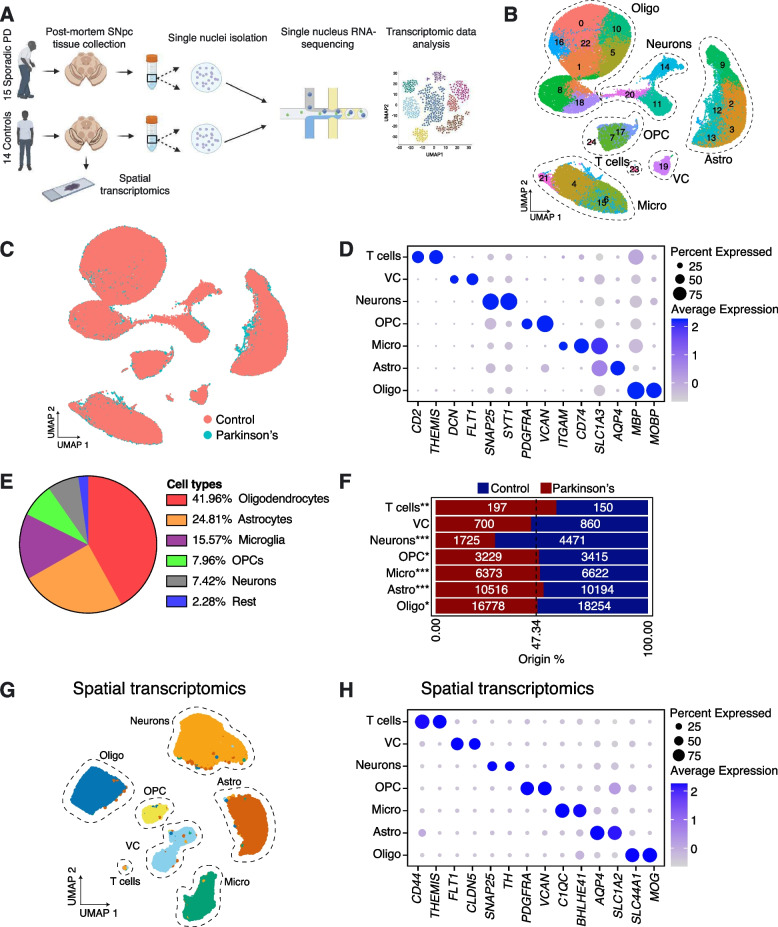
Cell types in human substantia nigra parscompacta and their susceptibility to PD. **A** Schematic of the experimental design. Nuclei were isolated from sections of frozen post-mortem brain containing substantia nigra pars compacta (SNpc) from Control (14) and PD (15) donors. Sequencing libraries were then prepared using the 10X Genomics Chromium platform and sent for standard Illumina sequencing. Spatial transcriptomics was performed on slices of fresh frozen SNpc tissue; samples were taken from 3 Control donors and 3 sporadic PD patients, selected from the cohort of 29 brains used for sequencing (Supplementary Table 1). **B** UMAP-based clustering of 83,484 high quality nuclei obtained from 15 PD and 14 Control samples. Clusters representing neurons, oligodendrocytes (Oligo), astrocytes (Astro), microglia (Micro), oligodendrocyte progenitor cells (OPC), T cells and vascular cells (VC) were identified, based on the expression of known marker genes (see also Supplementary Fig. 1A). **C** UMAP showing how nuclei from PD and Control samples distribute across the different clusters. **D** Expression of cell markers used to identify higher level cell types in SNpc. **E** A pie chart showing the percentage of major cell types in our SNpc dataset. **F** Bar plots showing the relative number of nuclei per cluster originating from Control or PD samples against their predicted abundance (based on 47.34% of all nuclei originating from PD samples: dashed line). A Binomial test was performed to see if there is any significant divergence of cell proportions from this value, * *p*-value < 0.05, ** *p*-value < 0.0005, *** *p*-value < 0.00005. **G** Independent clustering of the spatial transcriptomics dataset. **H** Confirmation of cell type marker expression using spatial transcriptomics

We sampled SNpc from post-mortem human brains of 15 sporadic Parkinson’s disease (PD) patients and 14 Control individuals (see Supplementary Table 1 for full pathology reports). Using a 10X Genomics Chromium platform, we performed single nucleus RNA-seq (snRNA-seq) on more than 80,000 high-quality nuclei from these 29 samples (Fig. 1A; Supplementary Table 2). UMAP dimensionality reduction on the merged PD and Control datasets showed that clustering was driven by inter-sample variability, as well as cell type identity. To control for inter-sample variability, we used an anchor-based integration approach to allow representative clustering and cell type identification. Louvain-based clustering identified 25 populations (Fig. 1B). Both PD and Control cells were found to be present within all cell clusters (Fig. 1C). We utilized a panel of markers to assign nuclei to various central nervous system (CNS) cell types, including neurons (*SYT1 & SNAP25*), astrocytes (*AQP4 & SLC1A3*), oligodendrocytes (*MOBP & MBP*), microglia (*CD74 & ITGAM*), vascular cells (VC) (*FLT1 & DCN*), oligodendrocyte progenitor cells (OPCs) (*VCAN & PDGFRA*) and T cells (*THEMIS & CD2*) (Fig. 1D; Supplementary Fig. 1A). We found that all the major cell types were detectable in multiple Control and PD samples (Supplementary Fig. 1B, Supplementary Table 2): 41.96% of all sampled cells were oligodendrocytes, with astrocytes and microglia representing 24.81% and 15.57% of the sample, respectively. We found that neurons made up 7.42% of cells, with OPCs at 7.96% and the remaining cell types representing 2.28% of the total cell population (Fig. 1E; Supplementary Table 3). These levels of cell recovery are consistent with other studies [6, 7], where cells were effectively captured at random from a single cell suspension and reinforce our belief that our dataset recapitulates the full range of cellular heterogeneity that can be captured using nuclei isolation and the 10X Chromium system (Supplementary Fig. 2)﻿.

As expected, evaluation of relative cell proportions showed that neurons are heavily depleted in PD samples, whereas the relative proportions of glial and T cells appear to increase (Fig. 1F). To confirm the presence of these cell types, we performed multiplexed spatial transcriptomics (Molecular Cartography by Resolve Biosciences), using a panel of cell type-specific markers (Supplementary Table 4). Clustering analysis of this data confirmed the presence of all the cell types detected by snRNA-seq (Fig. 1G, H).

### Neuronal subpopulations in aged human SNpc and their response in sporadic PD

**Fig. 2:**
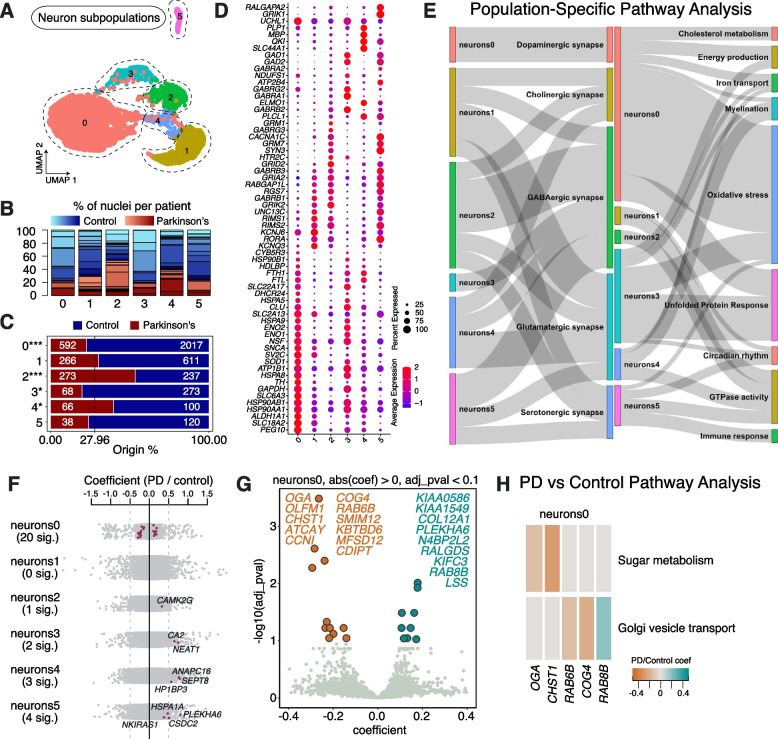
Neuronal subpopulations found in human substantia nigra pars compacta and their response to PD. **A** Re-clustering of neurons identified in Fig. 1B. **B** Proportion of nuclei coming from Control (blue) or PD (red) donors per neuronal type reported in Fig. 2A. Different shades of blue and red represent different donors. **C** A comparison of nuclei number (expressed as a percentage) deriving from PD or Control brains against their predicted abundance (based on 27.96% of all nuclei originating from PD samples: dashed line). A Binomial test was performed to see if there is any significant divergence of cell proportions from this value, * *p*-value < 0.05, ** *p*-value < 0.0005, *** *p*-value < 0.00005. **D** Selected marker genes for neuronal populations reported in Fig. 2A. The full list of marker genes is reported in Supplementary Table 5. **E** A summary of pathway over-representation analysis using the marker genes defining neuronal populations 0–5. Two Sankey diagrams are shown which illustrate the relationships between the various neuronal subpopulations and relevant cellular pathways identified (listed in Supplementary Table 6 in the column labeled "Group"). The thickness of the grey interconnecting lines is proportional to the number of individual pathways falling within a particular functional group that is significantly over-represented among the markers of a given cellular subpopulation, normalized to the number of pathways displayed per Sankey plot. **F** A stripe chart reporting the number of significantly up- or downregulated genes in nuclei originating from PD donors (compared to Controls) per neuronal subpopulation, found by fitting a linear mixed model. Red dots correspond to genes that are up- (coefficient > 0) or downregulated (coefficient < 0), with an adjusted *p*-value < 0.1 (ANOVA test with Benjamini–Hochberg correction). The full list of the up- and downregulated genes is reported in Supplementary Table 7. **G** A volcano plot reporting genes up- (cyan) or downregulated (orange) in nuclei originating from PD donors (compared to Controls) and assigned to subpopulation Neurons0. **H** Pathways over-represented by up- or downregulated genes in nuclei originating from PD donors (compared to Controls) and assigned to subpopulation Neurons0, as reported in Supplementary Table 8

To investigate PD-affected neuronal cell types, we re-clustered the neuronal population, revealing 6 distinct subpopulations, characterized by unique gene expression patterns, which we termed Neurons0—Neurons5 (Fig. 2A). All subpopulations were present in multiple PD and Control samples (Fig. 2B, Supplementary Table 2). Importantly, however, the numbers of cells in Neurons0 and Neurons3 were significantly lower in PD samples compared to Controls, whereas Neurons2 and Neurons4 were significantly over-represented in PD samples (Fig. 2C). To characterize these neuronal populations, we performed pathway over-representation analysis on the full list of marker genes for the various subpopulations (significantly enriched genes with ln-fold change > 0.25 and adjusted *p*-value < 0.05; Supplementary Table 5).

We found Neurons0 to be characterized by key markers associated with dopaminergic neurons, including *TH, SLC6A3, SNCA*, and *ALDH1A1* (Fig. 2D), highlighting that the primary subpopulation lost in PD samples is DA neurons. We also observed the expression of other dopaminergic markers, such as *SLC18A2* and *KCNJ6* (Fig. 2D). Paternally expressed gene 10 (*PEG10*), a DNA-binding protein coding gene, was another notable highly expressed cell marker [9] (Fig. 2D). We also observed high expression of *AGTR1* in this population (Supplementary Fig. 3), which has previously been linked to a subpopulation of DA neurons highly vulnerable to PD [8]. Pathway analysis of subpopulation marker genes showed over-representation of key cellular processes known to be implicated in PD pathology, such as energy production (e.g., *ATP1B1, ENO1* and *ENO2*), cholesterol metabolism (e.g., *DHCR24, CYB5R3* and *HDLBP*), iron transport (e.g., *FTL, FTH1* and *SLC22A17*), oxidative stress (e.g., *CHCHD10, CLU* and *SOD1*) and transcripts linked to the UPR (including chaperones, e.g., *HSPA8* and *HSP90AA1*) (Fig. 2D, E; Supplementary Fig. 3; Supplementary Table 6). Next, we examined differentially expressed genes (DEG) between PD and Control samples for this population of DA neurons and found 20 significant DEGs (Fig. 2F; Supplementary Table 7). Pathway analysis on up- or downregulated genes (PD vs Control) within this population suggests perturbations in sugar/glucose metabolism, due to the downregulation of O-GlcNAcase (*OGA*) and carbohydrate sulfotransferase 1 (*CHST1*), which is reported to be an early event in sporadic PD [10] (Fig. 2G, H; Supplementary Table 8). *KBTBD6*, involved in proteosome-mediated ubiquitin-dependent protein catabolic processes, was also downregulated, while dysregulation of the genes encoding Ras-related *RAB6B*, *RAB8B* and Component of Oligomeric Golgi Complex 4 (*COG4*) is consistent with dysfunction of Golgi to endoplasmic reticulum (ER) vesicle trafficking (Fig. 2G, H; Supplementary Table 8). These results are in line with recent reports of disrupted vesicle trafficking in PD, caused by the accumulation of alpha-synuclein, and the potential of RAB GTPases to rescue neurons from death [11–13].

Neurons3 represents a second neuronal population, which was found to be significantly depleted in PD samples. We found that cells in this population were characterized by the expression of key GABAergic markers, such as *GAD1, GAD2, GABRA1* and *GABRB2,* indicating a putative inhibitory identity (Fig. 2D, Supplementary Table 5). This population also expressed members of the heat shock protein family (*HSPA* and *HSP90*), as well as genes associated with dopamine secretion/metabolic processes/transport (e.g., *SYT11, KCNA2* and *ABAT*) (Supplementary Table 6). In addition, Neurons3 and Neurons0 both express markers associated with the UPR, oxidative stress, energy production and iron transport (Fig. 2E; Supplementary Table 6), suggesting underlying mechanisms for the shared vulnerability to cell death in PD. Differential gene expression analysis between PD and Control samples for Neurons3 showed upregulation of *NEAT1* and *CA2* (Fig. 2F; Supplementary Table 7). *NEAT1* encodes a long non-coding RNA previously shown to be upregulated in PD [14]. Elevated *CA2* levels in mitochondria have been associated with aging and neurodegeneration [15, 16].

We also explored the gene expression profiles of Neurons2 and Neurons4, which were both over-represented in PD samples (Fig. 2C). We observed the strongest expression of *SLC44A1*, the gene encoding the choline transporter-like protein 1, in Neurons4, whereas Neurons2 had the highest expression of serotonin 5-HT-2C receptor (*HTR2C*) (Fig. 2D). In contrast to PD-depleted dopaminergic Neurons0 and GABAergic Neurons3, the markers of the remaining neuronal populations do not show over-representation of processes related to the UPR, but do show enrichment in GTPase activity (Fig. 2E). Calmodulin-dependent protein kinase II gamma (*CAMK2G*) was differentially upregulated in PD Neurons2, while we found *ANAPC16*, *HP1BP3* and *SEPT8* differentially upregulated in PD Neurons4, compared to Controls (Fig. 2F; Supplementary Table 7).

### Astrocyte subpopulations in aged human SNpc and their responses in sporadic PD

**Fig. 3:**
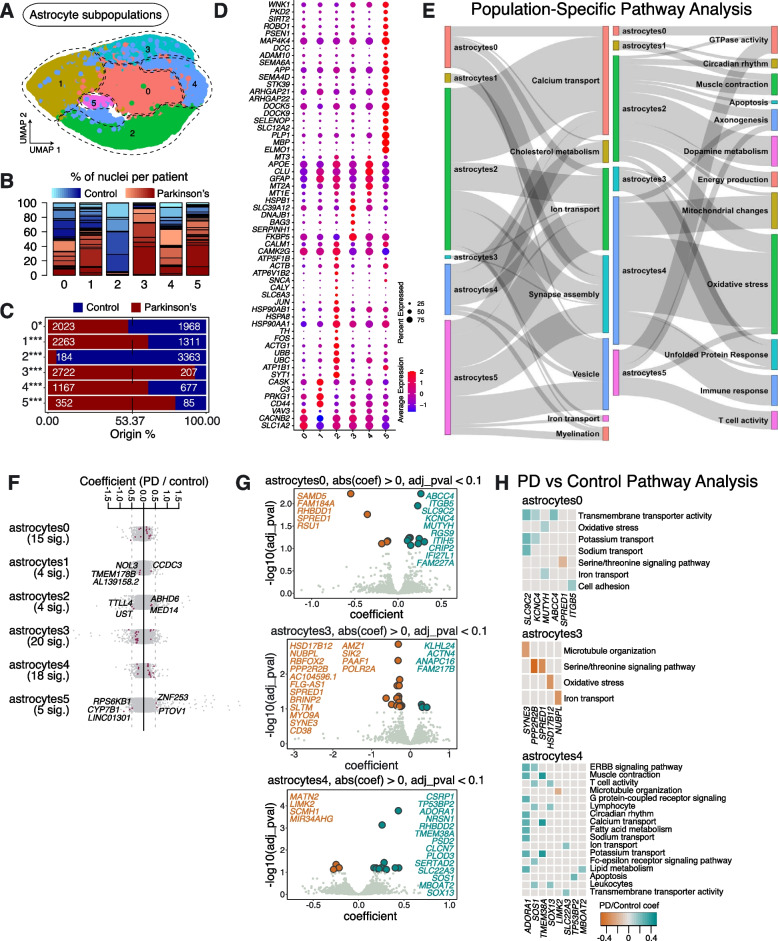
Astrocyte subpopulations found in human substantia nigra pars compacta and their response to PD. **A** Re-clustering of astrocytes identified in Fig. 1B. **B** Proportion of nuclei coming from Control (blue) or PD (red) patients per subpopulation reported in Fig. 3A. Different shades of blue and red represent different donors. **C** A comparison of nuclei number (expressed as a percentage) deriving from PD or Control brains against their predicted abundance (based on 53.37% of all nuclei originating from PD samples: dashed line). A Binomial test was performed to see if there is any significant divergence of cell proportions from this value, * *p*-value < 0.05, ** *p*-value < 0.0005, *** *p*-value < 0.00005. **D** Selected marker genes for astrocyte subpopulations reported in Fig. 3A. The full list of marker genes is reported in Supplementary Table 5. **E** A summary of pathway over-representation analysis using the marker genes defining astrocyte populations 0–5. Two Sankey diagrams are shown which illustrate the relationships between the various astrocyte subpopulations and relevant cellular pathways identified (listed in Supplementary Table 6 in the column labeled “Group”). The thickness of the grey interconnecting lines is proportional to the number of individual pathways falling within a particular functional group that is significantly over-represented among the markers of a given cellular subpopulation, normalized to the number of pathways displayed per Sankey plot. **F** A stripe chart reporting the number of significantly up- or downregulated genes in nuclei originating from PD donors (compared to Controls) per astrocyte subpopulation, found by fitting a linear mixed model. Red dots correspond to genes that are up- (coefficient > 0) or downregulated (coefficient < 0), with an adjusted *p*-value < 0.1 (ANOVA test with Benjamini–Hochberg correction). The full list of genes up- and downregulated in the various astrocyte subpopulations isolated from PD patients is reported in Supplementary Table 7. **G** A volcano plot reporting genes up- (cyan) or downregulated (orange) in nuclei originating from PD donors (compared to Controls): the astrocyte populations showing the most significant responses (Astrocytes0, Astrocytes3 and Astrocytes4) are shown. **H** Pathways over-represented by up- or downregulated genes in nuclei originating from PD patients (compared to Controls) and assigned to subpopulations Astrocytes0, Astrocytes3 and Astrocytes4, as reported in Supplementary Table 8

Extracting and re-clustering the astrocytes revealed 6 subpopulations, which we termed Astrocytes0—Astrocytes5 (Fig. 3A). All subpopulations were represented in multiple PD and Control samples (Fig. 3B, Supplementary Table 2). However, the numbers of cells in Astrocytes1, Astrocytes3, Astrocytes4 and Astrocytes5 were significantly over-represented in PD samples, while the number of cells in Astrocytes2 was significantly lower in PD samples compared to Controls (Fig. 3C).

Marker gene analysis revealed that Astrocytes2 showed enrichment for key genes linked to dopamine metabolism, including *SLC6A3, SNCA*, and importantly *TH*, suggesting that the vulnerability of *TH* enriched neurons in PD may be extended to other cell types (Fig. 3D, E). We also observed that the Astrocytes2 subpopulation expressed transcripts involved in ubiquitination (e.g., *UBB* and *UBC*), as well as transcripts associated with endocytic vesicle trafficking, protein folding (e.g., *HSP90AA1*, *HSP90AB1* and *HSPA8*), and *JUN* & *FOS* signaling, suggesting activation of apoptosis (Fig. 3D, E; Supplementary Fig. 4; Supplementary Tables 5, 6).

We observed that the Astrocytes3 subpopulation was mostly found in patients diagnosed with PD, suggesting that this astrocyte state may be PD-specific (Fig. 3C). Pathway over-representation analysis, using the specific marker genes identified for this subpopulation, identified pathways predominantly associated with the metabolism of fatty acids (e.g., *PTGES3*, *ABHD3*, *ADIPOR2* and *ABHD2*) and the UPR (e.g., *BAG3*, *SERPINH1*, *DNAJB1* and *HSPB1*) (Fig. 3D, E; Supplementary Table 6), suggesting a reactive-astrocyte identity [17]. Differential gene expression analysis between PD and Controls showed downregulation of genes involved in the serine-threonine signaling cascade (*PPP2R2B* and *SPRED1*), and the upregulation of genes associated with the ubiquitin ligase complex (*ANAPC16* and *KLHL24*) (Fig. 3G, H; Supplementary Tables 7, 8).

Pathway analysis on the cell markers defining Astrocytes5 highlighted biological processes including axon development, axon ensheathment, development of neuronal projections and synapse organization, suggesting that this subpopulation has an active role in neuronal maintenance and survival (Supplementary Table 6). In PD samples, differential gene expression analysis showed downregulation of *CYP7B1*, a gene involved in lipid homeostasis, and upregulation of *PTOV1*, a gene known to promote cell proliferation, in this subpopulation [18] (Fig. 3F; Supplementary Table 7).

The well-known markers of reactive astrocytes, *C3* and *CD44*, are more enriched in Astrocytes1, a subpopulation likely to represent another reactive astrocyte state [19–21] (Fig. 3D). Genes involved in glutamate metabolism and synapse assembly were also highly expressed in this astrocyte population (Fig. 3E; Supplementary Table 6). Pathway analysis on the DEGs in Astrocytes1 between PD and Control samples indicated the dysregulation of tumor necrosis factor (TNF)-mediated signaling (*CCDC3* and *NOL3*) in PD (Fig. 3F; Supplementary Table 7).

Astrocytes4 was enriched in PD samples and expressed high levels of various metallothionein genes (e.g., *MT2A*, *MT1E* and *MT3*) (Fig. 3D). This astrocyte subpopulation also highly expresses *GFAP*, a canonical marker of astrogliosis [22] (Fig. 3D). Astrocytes4 also has the highest expression of *APOE*, *MT3* and *CLU*, which are associated with mitochondrial changes, oxidative stress and immune response-related processes (Fig. 3D, E; Supplementary Fig. 4). At the level of differential gene expression between cells obtained from PD and Control samples, we found upregulation of T cell activity, lipid metabolism and ion transport in PD samples compared to Controls, in this subpopulation (Fig. 3H; Supplementary Table 8).

### Microglia subpopulations in aged human SNpc and their response in sporadic PD

**Fig. 4:**
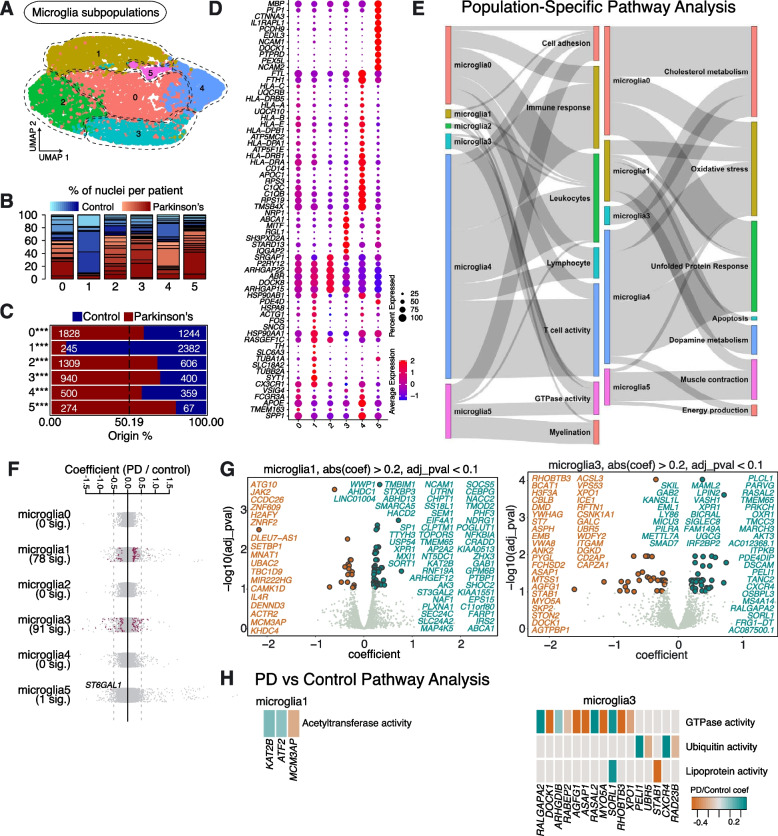
Microglia subpopulations found in human substantia nigra pars compacta and their response to PD. **A** Re-clustering of microglia identified in Fig. 1B. **B** Proportion of nuclei coming from Control (blue) and PD (red) patients per subpopulation reported in Fig. 4A. Different shades of blue and red represent different donors. **C** A comparison of nuclei number (expressed as a percentage) deriving from PD or Control brains against their predicted abundance (based on 50.19% of all nuclei originating from PD samples: dashed line). A Binomial test was performed to see if there is any significant divergence of cell proportions from this value, * *p*-value < 0.05, ** *p*-value < 0.0005, *** *p*-value < 0.00005. **D** Selected marker genes for microglial populations shown in Fig. 4A. The full list of marker genes is reported in Supplementary Table 5. **E** A summary of pathway over-representation analysis using the marker genes defining microglia populations 0–5. Two Sankey diagrams are shown which illustrate the relationships between the various microglia subpopulations and relevant cellular pathways identified (listed in Supplementary Table 6 in the column labeled “Group”). The thickness of the grey interconnecting lines is proportional to the number of individual pathways falling within a particular functional group that is significantly over-represented among the markers of a given cellular subpopulation, normalized to the number of pathways displayed per Sankey plot. **F** A stripe chart reporting the number of significantly up- or downregulated genes in nuclei originating from PD donors (compared to Controls) per microglial subpopulation, found by fitting a linear mixed model. Red dots correspond to genes that are up- (coefficient > 0) or downregulated (coefficient < 0), with an adjusted *p*-value < 0.1 (ANOVA test with Benjamini–Hochberg correction). The full list of genes up- or downregulated in the various microglia subpopulations isolated from PD patients is reported in Supplementary Table 7. **G** Volcano plots reporting genes up- (cyan) or downregulated (orange) in nuclei originating from PD donors (compared to Controls): two microglia populations (Microglia1 and Microglia3) are shown. **H** Pathways over-represented by up- or downregulated genes in nuclei from PD patients (compared to Controls) and assigned to subpopulations Microglia1 and Microglia3, as reported in Supplementary Table 8

We next explored the molecular profiles of microglia subpopulations affected in sporadic PD. Re-clustering analysis of microglia revealed 6 subpopulations, which we named Microglia0—Microglia5 (Fig. 4A). All subpopulations were represented in multiple PD and Control samples (Fig. 4B, Supplementary Table 2).

As with astrocytes, we saw that one population, Microglia1, was significantly depleted in samples from patients diagnosed with sporadic PD (Fig. 4C). Marker gene analysis again revealed that this was the only microglia population enriched in markers involved in dopamine metabolism, including *TH* (Fig. 4D, E; Supplementary Fig. 5; Supplementary Tables 5, 6). Furthermore, this population expresses a high number of transcripts linked to processes involved in the UPR (e.g., *HSP90AA1*, *HSP90AB1* and *HSPA8*), like we observed for Astrocytes2 (Fig. 4D, E). Similarly, Microglia1 was also positive for markers associated with apoptosis (e.g., *FOS* and *ACTG1*) (Fig. 4D, E). A high number of differentially expressed genes was observed between PD and Control samples (Fig. 4F; Supplementary Table 7). Of particular interest, Microglia1 shows upregulated expression of acetyltransferase activity (*KAT2B*, *ATF2* and *MCM3AP*) in PD, which is known to be involved in inflammatory responses in microglia [23] (Fig. 4H; Supplementary Table 8).

Among the markers of Microglia2, *P2RY12* stands out as a P2Y receptor involved in microglial motility and migration towards (damaged) cells releasing ATP, an initiating event in neuroinflammation [24] (Fig. 4D). Furthermore, *DOCK8*, a neuroinflammation-associated gene, was also highly expressed in Microglia2 (Fig. 4D), which also expressed ARHGAP family transcripts (including *ARHGAP22* and *ARHGAP15*), which are known to be linked to alterations in the microglial activation state upon aging [25, 26]. Together, this suggests that Microglia2 may represent a pro-inflammatory subset of microglia, central to the neuroinflammatory response seen in PD.

Microglia4 was characterized by the high expression of *APOE* and *SPP1* (Fig. 4D). Microglia4 is the only subpopulation of microglia that expressed a high level of *APOE* (Fig. 4D). Microglia4 also appears to represent a population of reactive microglia, expressing genes involved in the complement cascade (e.g., *C1QC*, *C1QB* and *C1QA*), the human leukocyte antigen (HLA) system (e.g., *HLA-DRA* and *HLA-DRB1*), the UPR (e.g., *HSP90* and *HSPA*), and the oxidative stress response (e.g., *HSPA1A*, *TREM2*, *GSTP1* and *HSPB1*) (Fig. 4D, E). Microglia2 and Microglia4 do not show significant differential expression of genes between PD and Control conditions (Fig. 4F; Supplementary Table 7).

Microglia3 was characterized by expression of *ABCA1*, *MITF* and *STARD13* (Fig. 4D). This subpopulation is the only microglial subpopulation that expressed high levels of *SRGAP1*, *SH3PXD2A* and *RGL1* (Fig. 4D). Differential gene expression analysis between PD and Controls showed downregulation of genes involved in GTPase activity (*DOCK1, AGFG1* and *ASAP1*), and dysregulation of ubiquitin activity (*PELI1* and *CXCR4*) (Fig. 4G, H; Supplementary Tables 7, 8).

### Oligodendrocyte subpopulations in aged human SNpc and their response in sporadic PD

**Fig. 5:**
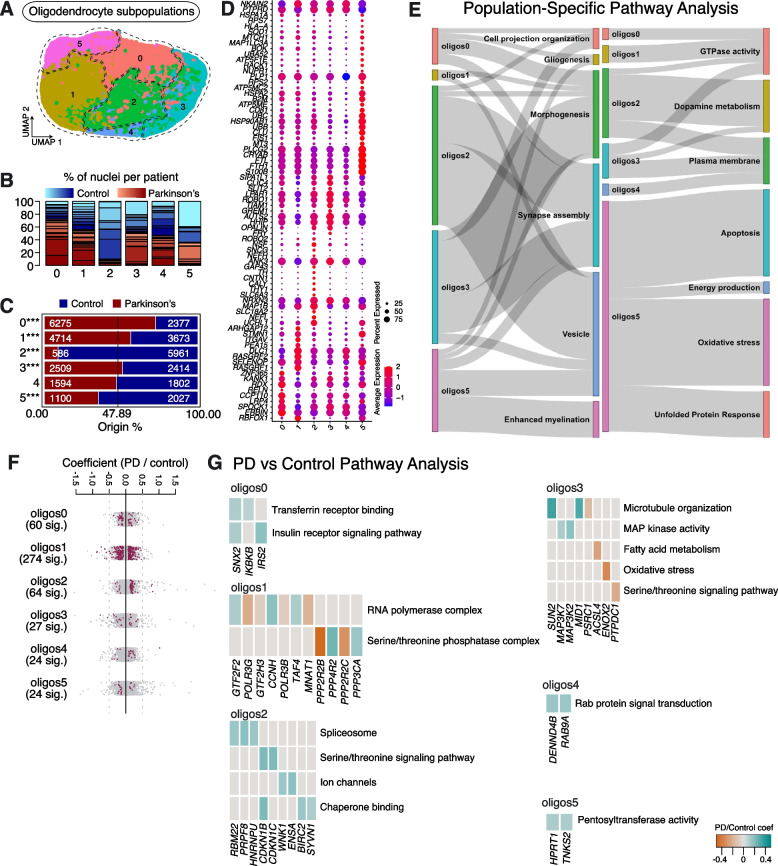
Oligodendrocyte subpopulations found in human substantia nigra pars compacta and their response to PD. **A** Re-clustering of oligodendrocytes identified in Fig. 1B. **B** Proportion of nuclei coming from Control (blue) and PD (red) patients per subpopulation reported in Fig. 5A. Different shades of blue and red represent different donors. **C** A comparison of nuclei number (expressed as a percentage) deriving from PD or Control brains against their predicted abundance (based on 47.89% of all nuclei originating from PD samples: dashed line). A Binomial test was performed to see if there is any significant divergence of cell proportions from this value, * *p*-value < 0.05, ** *p*-value < 0.0005, *** *p*-value < 0.00005. **D** Selected marker genes for oligodendrocyte subpopulations shown in Fig. 5A. The full list of marker genes is reported in Supplementary Table 5. **E** A summary of pathway over-representation analysis using the marker genes defining oligodendrocyte populations 0–5. Two Sankey diagrams are shown which illustrate the relationships between the various oligodendrocyte subpopulations and relevant cellular pathways identified (listed in Supplementary Table 6 in the column labeled “Group”). The thickness of the grey interconnecting lines is proportional to the number of individual pathways falling within a particular functional group that is significantly over-represented among the markers of a given cellular subpopulation, normalized to the number of pathways displayed per Sankey plot. **F** A stripe chart reporting the number of significantly up- or downregulated genes in nuclei originating from PD donors (compared to Controls) per oligodendrocyte subpopulation, found by fitting a linear mixed model. Red dots correspond to genes that are up- (coefficient > 0) or downregulated (coefficient < 0), with an adjusted *p*-value < 0.1 (ANOVA test with Benjamini–Hochberg correction). The full list of genes up- or downregulated in the various oligodendrocyte subpopulations isolated from PD patients is reported in Supplementary Table 7. **G** Pathways over-represented by up- or downregulated genes in nuclei originating from PD donors (compared to Controls) and assigned to the oligodendrocyte subpopulations, as reported in Supplementary Table 8

We also performed re-clustering on the oligodendrocyte population, revealing 6 subpopulations, which we named Oligos0 – Oligos5 (Fig. 5A). All subpopulations were represented in multiple PD and Control samples (Fig. 5B, Supplementary Table 2). However, the numbers of cells in Oligos2 and 5 were significantly lower in PD samples compared to Controls, whereas Oligos0, Oligos1 and Oligos3 were significantly enriched in PD. Oligos4 was not significantly changed between PD and Control (Fig. 5C).

Like Astrocytes2 and Microglia1, Oligos2 represents a population enriched for *TH, SLC6A3* and *SNCG* (genes associated with dopamine metabolism) and it is largely depleted in sporadic PD samples (Fig. 5C, D, E). This population is also enriched in transcripts linked to axon development and synapse organization (e.g., *UCHL1, NEFL, MAP1B* and *NRXN3*), transcripts linked to ion transport (e.g., *CNTN1* and *ANK3*) and the synaptic vesicle cycle (e.g., *SLC18A2* and *CALY*) (Fig. 5D, E; Supplementary Fig. 6; Supplementary Tables 5, 6). Interestingly, unlike *TH* enriched astrocytes and microglia, this *TH* enriched oligodendrocyte population does not show enrichment for genes involved in unfolded protein or oxidative stress responses (Fig. 5E). All oligodendrocyte subpopulations show a strong response in differential gene expression between PD and Control samples (Fig. 5F; Supplementary Table 7). Among them, Oligos2 demonstrates upregulation of genes linked to spliceosome function (*RBM22, PRPF8* and *HNRNPU*), ion channel activity (*WNK1* and *ENSA*), serine/threonine kinase activity (*CDKN1B* and *CDKN1C*) and chaperone binding (*BIRC2* and *SYVN1*) (Fig. 5G, Supplementary Tables 7, 8). Moreover, Oligos2 also shows up-regulation of *CAMK2G*, consistent with the dysregulation of calcium homeostasis and alterations in calmodulin-dependent protein kinase signaling observed in PD [27] (Supplementary Table 7). In addition, expression of the translocase of outer mitochondrial membrane 40 gene (*TOMM40*) was also dysregulated (Supplementary Table 7). The protein encoded by this gene is localized in the outer membrane of mitochondria and is the channel-forming subunit of a translocase complex essential for the import of protein precursors [28].

The second population of oligodendrocytes depleted in sporadic PD is Oligos5 (Fig. 5C). Oligos5 highly expresses *CRYAB*, a small heat shock protein, which is implicated in various protein aggregation-related neurodegenerative diseases, such as PD, Alzheimer’s disease (AD), amyotrophic lateral sclerosis (ALS) and prion disorders [29] (Fig. 5D). Like Microglia4, Oligos5 has high expression of *FTL* and *FTH1*, genes which encode proteins involved in iron storage (Fig. 5D). Oligos5 also highly expresses *S100B*, which has been associated with the glial stress response in the midbrain of PD patients [6] (Fig. 5D). Pathway over-representation analysis on the markers of this population shows enrichment of terms related to oxidative stress (e.g., *CRYAB, MT3, SELENOP* and *MAP1LC3A*), the response to protein aggregates (e.g., *CLU, HSPA2, HSPA1A* and *HSP90AB1*), ATP biosynthesis (e.g., *ATP5ME, ATP5F1E* and *ATP5MC2*), mitochondrial function (e.g., *MT3, UBB, UBC* and *UBA52*) and apoptosis (e.g., *FIS1, UBB, RACK1, RPS3* and *NUPR1*) (Fig. 5D, E; Supplementary Fig. 6; Supplementary Tables 5, 6). At the differential gene expression level, Oligos5 isolated from PD samples shows upregulation of hypoxanthine guanine phosphoribosyltransferase (*HPRT1*) and TRF1-interacting ankyrin-related ADP-ribose polymerase 2 (*TNKS2*), which are both involved in regulating pentosyltransferase activity (Fig. 5G; Supplementary Table 8).

Oligos1 is more highly represented in PD samples than Controls and highly expresses *RBFOX1*, a dosage-sensitive gene whose disruption is associated with neurodevelopmental conditions and synaptic transmission, through its critical role in the control of mRNA splicing [30] (Fig. 5D). Oligos1 does not show strong enrichment for transcripts linked to myelination, vesicle trafficking or synapse assembly processes (Fig. 5E). Between PD and Control conditions, differential gene expression analysis identifies genes associated with GO/KEGG terms including RNA polymerase complex function (e.g., *GTF2F2, POLR3G, GTF2H3* and *CCNH*), serine/threonine phosphatase activity (e.g., *PPP2R2B, PPP4R2, PPP2R2C* and *PPP3CA*), histone acetyltransferase complex function (e.g., *CREBBP, EP300, JADE2* and *TAF4*), protein ubiquitination (e.g., *ANAPC16, FBXW8, SKP2* and *KLHL21*) and lysosomal activity (e.g., *DMXL1, ARSB* and *SORT1*) (Fig. 5G; Supplementary Table 8).

Oligos3 is another oligodendrocyte subpopulation that is significantly over-represented in PD samples (Fig. 5C). Oligos3 expresses high levels of *OPALIN*, a marker of myelinating oligodendrocytes [6] (Fig. 5D). Pathway analysis on marker genes for Oligos3 shows strong enrichment for transcripts involved in synapse assembly (Fig. 5E). At the level of differential gene expression, various transcripts in Oligos3 showed either upregulation or downregulation in PD samples, when compared to Controls. Upregulated genes include several involved in MAP kinase activity (*MAP3K7* and *MAP3K2*) and microtubule organization (*SUN2* and *MID1*); downregulated genes include those involved in fatty acid metabolism (*ACSL4*), oxidative stress (*ENOX2*) and the serine/threonine signaling pathway (*PTPDC1*) (Fig. 5G; Supplementary Table 8).

### PD-associated genes and genes near PD-associated variants show cell type-specific expression patterns

Genetic factors play a role in the development of Parkinson's disease. Although genetic methods and population studies, including genome-wide association studies (GWAS), have identified various monogenic variants and susceptibility loci for PD, it remains to be shown whether these monogenic PD genes, or genes-associated with these loci, are specific to SNpc cell types (or their subpopulations).

**Fig. 6:**
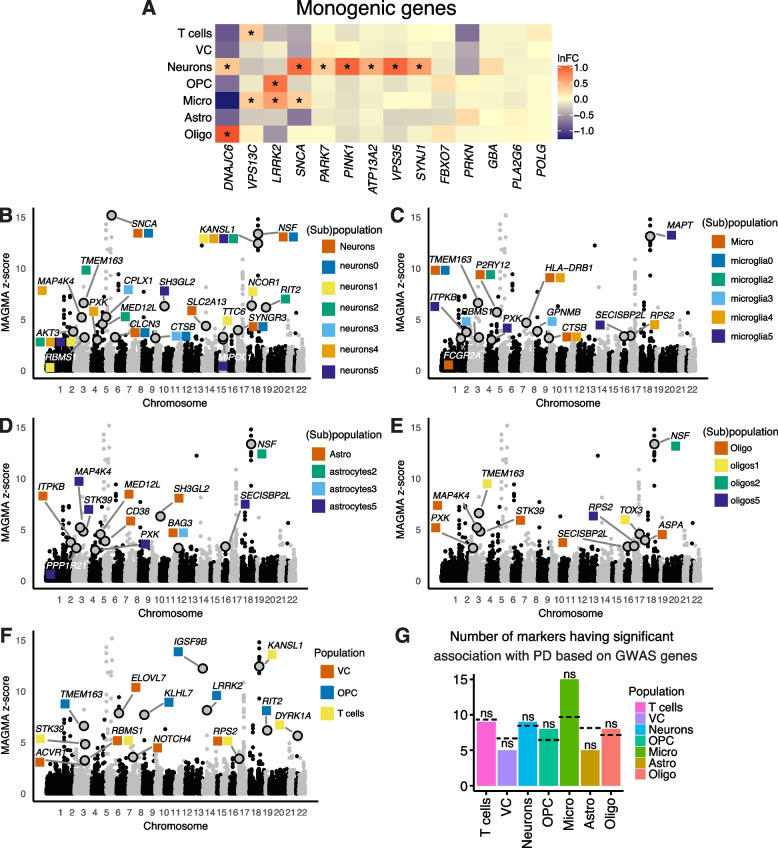
PD-associated genes and genes near PD-associated variants show cell type-specific expression patterns. **A** Cell type enriched expression of high-confidence genes associated with monogenic PD. Ln-fold change of SCT-normalized counts is shown (Wilcoxon test, *FDR-corrected *p*-value < 0.05; see Supplementary Table 5). **B**-**F** Pseudo-Manhattan plots of the genes near PD-associated variants generated by MAGMA on the GWAS dataset curated by Nalls and colleagues [32]. The top differentially expressed genes per population/subpopulation which show significant association with PD variants (MAGMA *p*-value lower than 0.001, ln-fold change > 0.3 for population-level analysis, and ln-fold change > 0.25 for subpopulation-level analysis) are shown; VC, vascular cells; OPC, oligodendrocyte progenitor cells; Micro, microglia; Astro, astrocytes; Oligo, oligodendrocytes. **G** The number of cell type enriched marker genes that are also near PD-associated variants. The results of two-sided Binomial tests are shown at the top (ns = non-significant). The dashed line shows the expected number of markers given the null hypothesis (see ‘Methods’ and Supplementary Table 9)

In our study, we first explored the cell type specificity of a set of high-confidence genes associated with monogenic PD reported in [31]. We observed that certain genes exhibit cell type-specific enrichment (Fig. 6A). For instance, *SNCA* showed enrichment in neurons and microglia, *LRRK2* in microglia and OPC, *DNAJC6* in neurons and oligodendrocytes, and *VPS13C* in microglia and T cells (Fig. 6A). Several monogenic PD genes demonstrated significant enrichment specifically in neurons, such as *PARK7, PINK1, ATP13A2, VPS35* and *SYNJ1* (Fig. 6A). Moreover, amongst all neuronal subtypes, most of the monogenic PD genes investigated show significant enrichment in the DA neuronal population Neurons0 (*DNAJC6, SNCA, PARK7, PINK1, ATP13A2, VPS35* and *SYNJ1*). Additionally, *DNAJC6* showed enrichment in Astrocytes5 and Microglia5, *SNCA* in Astrocytes2 and Astrocytes5, and *VPS35* in Neurons3. In contrast, *PRKN* shows enrichment in all neuronal subpopulations except Neurons0 and Neurons3 (Supplementary Fig. 7A). Furthermore, we were able to confirm some of these findings using spatial transcriptomics on independent tissue sections obtained from the same donors sampled in our snRNA-seq analysis, such as *SNCA* and *ATP13A2* enrichment in neurons and *LRRK2* enrichment in microglia (Supplementary Fig. 7B, Supplementary Table 5). However, no significant enrichment of *LRRK2* in OPC was observed, possibly due to the small size of the OPC population detected in our spatial data.

Although the caveats of GWAS studies are increasingly recognized, we attempted to identify genes near PD-associated variants in our data by cross-referencing to the largest PD GWAS dataset publicly available at the time we conducted our study [32]. This dataset, curated by Nalls and colleagues, contains data collected over 17 studies, and reports 7.8 million single nucleotide polymorphisms (SNPs) identified in 37,688 diagnosed PD cases, 18,618 proxy-cases (individuals who do not have PD but have a first degree relative who does) and 1.4 million Controls. We predicted 203 genes to be highly associated with GWAS SNPs using Multi-marker Analysis of GenoMic Annotation (MAGMA) analysis (Supplementary Table 9). In this analysis, we also observed an overlap of MAGMA identified genes with the markers of distinct cell types we identified in our SNpc dataset (Fig. 6B-F, Supplementary Fig. 7C). Specifically, neuronal cell markers exhibited an overlap with genes near PD-associated variants in GWAS, such as *SNCA;* microglia cell markers with *P2RY12* and *LRRK2;* and OPC cell markers with *LRRK2* (Fig. 6B, C, F; Supplementary Table 9)*.* We further explored the association of genes near PD-associated variants at the subpopulation level and found that several subpopulations also displayed association with these genes. For instance, PD depleted Neurons0 and Microglia2 exhibited an association with *SNCA* and *P2RY12*, respectively* (*Fig. 6B, C). Binomial testing did not reveal significant enrichment of the GWAS PD-associated-variant-proximal genes in any specific cell type (Fig. 6G), which could be due to the inherent uncertainty in assigning causal genes to GWAS lead variants. Overall, our analyses reveal that cell (sub)populations show an association with at least a handful of genes near PD GWAS SNPs, as identified through MAGMA analysis, and display enrichment in several monogenic PD genes, consistent with a complex interplay between cells contributing to disease pathogenesis.

### *TH* among several common genes enriched in cell types depleted in sporadic PD samples

**Fig. 7:**
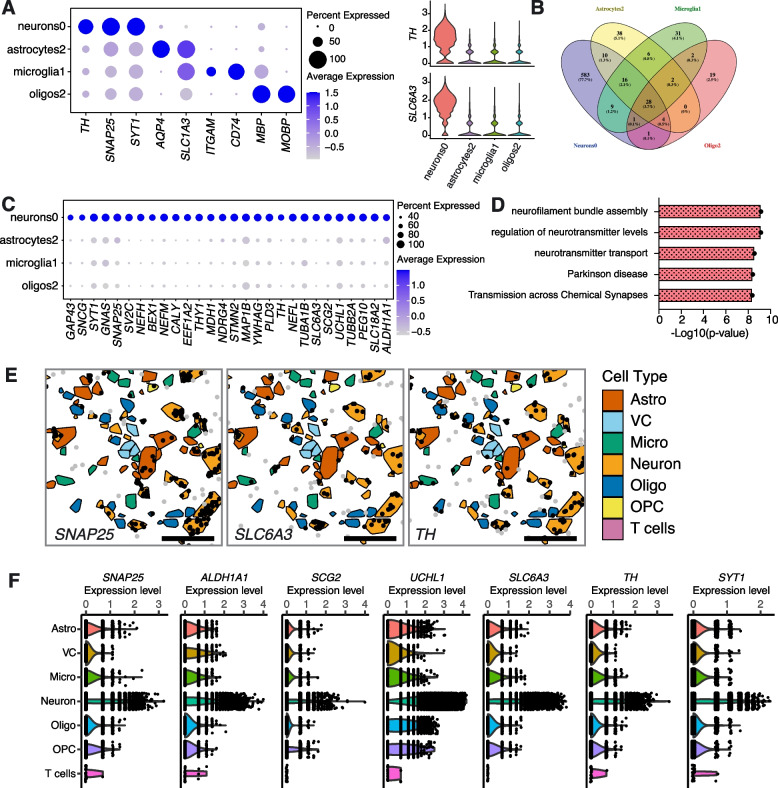
Molecular similarities between tyrosine hydroxylase (*TH*) enriched cell types depleted in the substantia nigra pars compacta of PD patients. **A** *TH* expression comparison between dopaminergic Neurons0 and *TH* enriched glial cell types (Astrocytes2, Microglia1 and Oligos2). Higher level cell markers were used to confirm the identity of these subpopulations. **B** Venn diagram showing overlap of cell markers defining dopaminergic Neurons0 and *TH* enriched glial cell types. **C** Gene expression levels of cell markers shared between dopaminergic Neurons0 and *TH* enriched glial cell types. **D** GO term pathway analysis on the shared cell markers. **E** Representative images from spatial transcriptomics experiments showing the presence of *SNAP25*, *SLC6A3* and *TH* transcripts (dots) in higher-order cell types (represented as polygons). Astro, astrocytes; VC, vascular cells; Micro, microglia; Oligo, oligodendrocytes; OPC, oligodendrocyte progenitor cells. Scale bar, 50 µm. **F** Gene expression levels for shared cell markers of higher-order cell types determined by spatial transcriptomics. Violin plots of SCT-normalized data are shown

Along with our analyses showing a strong depletion of *TH* enriched neurons in sporadic PD, we also tentatively identified *TH* enriched astrocyte, microglia and oligodendrocyte subpopulations that appear depleted in PD. To try and increase the robustness of this finding we took two independent approaches. First, we analysed the largest snRNA-seq dataset currently available of human substantia nigra tissue (obtained from cohorts of Controls and PD patients independent of our study), which was published by Kamath and colleagues [8]. Analysis revealed that although the correspondence of subpopulations detected in the individual datasets was not entirely comparable (Supplementary Figs. 8, 9, 10), it was possible not only to detect the presence of *TH*-positive (*TH* +) glia in the Kamath et al. dataset but also observe that these cells were depleted in samples from PD donors (Supplementary Figs. 8C, 9C, 10C). To investigate possible shared molecular mechanisms of action, we compared the gene expression profiles of the four *TH* enriched subpopulations. First, we compared the levels of *TH* expression across these cell types and observed that *TH* enriched glial cells expressed *TH* at a relatively low level compared to DA neurons (Fig. 7A; Supplementary Fig. 11). We re-confirmed the identity of these subpopulations using a panel of higher-level cell type markers. For example, Oligos2 is enriched in oligodendrocyte markers (*MBP* and *MOBP*) and *TH* (Fig. 7A). Furthermore, we also observed a relatively lower percentage of the glia populations expressing *TH* compared to dopaminergic Neurons0 (Fig. 7A). We next identified 28 cell markers that are shared between the depleted *TH* enriched cell types (Fig. 7B, C; Supplementary Table 10). We compared the expression of these genes across the PD depleted *TH* enriched subpopulations and again found them to be expressed at lower levels in glia compared to dopaminergic neurons (Fig. 7C). GO term pathway analysis on these shared genes demonstrated enrichment in pathways involved in dopaminergic neurogenesis (e.g., *ALDH1A1*, *SLC6A3*, *SLC18A2* and *TH*), neurofilament assembly (e.g., *NEFM, NEFH* and *NEFL*), regulation of neurotransmitter levels and transport (e.g., *SLC6A3, SLC18A2, SNAP25* and *SNCG*) and synaptic signalling (*MAP1B* and *YWHAG*) (Fig. 7D, Supplementary Table 10). We confirmed *TH* expression in all major cell types resident in the SNpc using spatial transcriptomics with single cell resolution (Molecular Cartography) on tissue sections obtained from Control and PD donors (see ‘Methods’). Consistent with the results obtained using single nucleus sequencing, we found that glial cells in general express lower amounts of *TH* transcripts compared to neurons (Fig. 7E, F).

## Discussion

Here we provide a new resource for PD research: a snRNA-seq dataset of human post-mortem SNpc samples, collected from 15 sporadic PD and 14 Control brains. Our dataset reports the transcriptomes of ~ 84K high-quality nuclei and allows us to identify PD-induced cell type-specific changes in all major cell types of human substantia nigra pars compacta. Oligodendrocytes (~ 41%) were the most abundant cell type in our dataset, followed by astrocytes (~ 25%), microglia (~ 15%), neurons (~ 7%), OPC (~ 8%) and T cells (< 2%). We found an increase in relative proportions of glial and T cells and a decrease in neurons in samples from patients diagnosed with sporadic PD. As expected, we observed a dropout of dopaminergic neurons (Neurons0) in PD patients. This population has higher expression of *AGTR1*, in line with previous reports [8, 33]. In addition, we also identified a population of GABAergic neurons (Neurons3), that shows a high vulnerability to PD (see below).

We further mined this transcriptome resource, to assess the expression patterns of genes associated with monogenic PD and PD GWAS variants across the various cell types. We observed that neurons are enriched in several monogenic PD genes when compared to other cell types, consistent with the known vulnerability of neurons in PD. Specifically, in line with the findings of previous single nucleus transcriptomics studies [6, 8], we observed robust enrichment of *SNCA* in neurons, *LRRK2* in OPC and microglia, *DNAJC6* enrichment in oligodendrocytes, and no cell type-specific enrichment of *PLA2G6* or *GBA.* At the subpopulation level, most genes showed enrichment in the dopaminergic neuronal population Neurons0. In addition to Neurons0, *DNAJC6* exhibited significant enrichment in Astrocytes5 and Microglia5, *SNCA* in Astrocytes5 and PD depleted Astrocytes2, and *VPS35* in Neurons3. Interestingly, *PRKN* showed enrichment in all neuronal subpopulations except for PD depleted Neurons0 and Neurons3. Cross-referencing the PD GWAS SNPs dataset curated by Nalls and colleagues [32] revealed that out of 203 genes (MAGMA *p*-value lower than 0.001) located near a relevant PD GWAS variant, 46 were enriched in several major cell populations, although no significant enrichment in any single population was observed. Additionally, some genes displayed cell subtype association. Notably, *P2RY12* was associated with microglia, consistent with a previous study [6], and specifically associated with Microglia2, a microglia subpopulation displaying markers associated with an early activation state [24]. The suggestion that the expression of genes near PD-associated genetic variants could drive cell type-specific, disease-relevant pathology provides a rationale for future studies on how such genetic variants exert their pathological functions using iPSC-derived models.

While we detected various classes of neurons, the major effect of PD appears to be on the dopaminergic system. Besides dopaminergic neurons, however, populations of astrocytes, microglia and oligodendrocytes also appear to be *TH* enriched and lost in PD at statistically significant levels, leading us to speculate that loss of dopaminergic signaling in PD has a complex multi-cellular basis. Dopaminergic neurons show high expression of the protein chaperones *HSPA8* and *HSP90AA1*, which are known to regulate protein folding and stability [34, 35]. GO/KEGG term over-representation analysis of genes up- or downregulated in PD samples (compared to Controls) suggests sugar metabolism and Golgi to ER vesicle trafficking as the main processes impacted within dopaminergic neurons. This is consistent with reports that glycosylation in the Golgi and ER are critical quality control steps in protein folding [36, 37]. Furthermore, N-acetyl-glucosamine modification makes alpha-synuclein less toxic for neurons. Taken together, these observations are consistent with glycosylation as a cellular mechanism reducing aggregation of misfolded alpha-synuclein within dopaminergic neurons [38]. We further speculate that abnormal glycosylation in PD patients may also be related to their abnormal sugar uptake [37, 39].

In PD research, the focus has been on degeneration of dopaminergic neurons and the functional loss of dopaminergic transmission. However, current evidence suggests that neurotransmitters such as GABA, glutamate, serotonin and acetylcholine may play a key role in the pathophysiology of PD. In our PD samples, GABAergic neurons3 appears significantly reduced compared to Control samples. In vivo, dopaminergic neuronal axons in the striatum release GABA. Hence, loss of dopaminergic neurons in PD could lead to a decreased level of GABA in basal ganglia circuits [40]. Interestingly, pharmacological GABA agonists have been shown to protect dopaminergic neurons and alleviate motor symptoms in animal models of PD [41, 42]. A recent study has shown that induced GABAergic neurons, generated in vivo from striatal astrocytes using a forward programming approach, ameliorate motor symptoms in a neurotoxin-induced murine model of PD [43].

In our PD samples, we found upregulation of the *NEAT1* gene, which encodes a long non-coding RNA in GABAergic Neurons3. There is contradicting evidence regarding upregulation of *NEAT1* as a protective or deleterious mechanism in PD. A recent report has shown increased levels of *NEAT1* in the peripheral blood cells of PD patients [44]. There are also studies showing that *NEAT1* is highly expressed in the substantia nigra of PD patients and may play a role in regulating mitochondrial stability [14]. Some reports suggest a role for *NEAT1* over-expression in inducing apoptosis and autophagy [45]. In contrast, some reports suggest *NEAT1* upregulation is associated with protection from oxidative stress and regulation of neuroinflammation [46, 47]. Since there are so many contradicting results from multiple studies, our data may provide some clarity on the role of *NEAT1* in PD.

Dysregulation of astrocyte functions has been implicated in many neurodegenerative diseases. We discovered four distinct astrocyte populations enriched in PD samples. Among them was an astrocyte population which displayed increased expression of genes associated with the UPR. A recent report has shown that the UPR can trigger a reactive state in astrocytes that can cause non-cell autonomous neurodegeneration, suggesting that this population of astrocytes may be directly toxic to neurons [17]. The Astrocytes4 population in PD showed high expression of *GFAP* and *APOE*, as well as transcripts linked to mitochondrial changes and oxidative stress. Astrocytic oxidative stress is thought to play an important role in the pathogenesis of both familial and sporadic PD [48]. Several studies have highlighted the role of mitochondria-induced oxidative stress in astrocytes in neurodegeneration [49, 50].

We explored microglia subpopulations in our dataset and their associated molecular profiles from both Control and PD samples. We observed a high degree of heterogeneity in disease-responsive subpopulations of microglia. All microglia subpopulations showed over-representation in PD samples, except *TH* enriched Microglia1. We found a microglia subpopulation (Microglia2) highly expressing a known marker of microglia, *P2RY12,* and several markers associated with neuroinflammation. However, this subpopulation does not show enrichment for any transcripts linked to an immune response, suggesting that it may represent an early stage of microglia activation. On the other hand, two microglia subpopulations (Microglia4 and Microglia0) showed enrichment for transcripts involved in the UPR, oxidative stress and cholesterol metabolism. Interestingly, Microglia4 displayed higher expression of genes associated with immune response compared to Microglia0. This suggests that Microglia0 may be a state forming a continuum with Microglia4, possibly arising due to the UPR and oxidative stress. Microglia4 showed the highest expression of iron storage genes (*FTL* and *FTH1*), *APOE*, human leukocyte antigen (HLA) genes and complement cascade components (*C1QC, C1QB* and *C1QA*). Higher expression of *HLA-DRA* in microglia has been reported in aged ventral tier substantia nigra tissue, using the MPTP animal model of PD [51]. Also, there have been reports of alpha-synuclein aggregate induced activation of the complement pathway and complement-mediated neurotoxicity [52].

Like the rest of the cell types, we detected a high degree of heterogeneity in the oligodendrocyte population. Oligos0 and Oligos1, major subsets of the oligodendrocyte population, which are highly over-represented in PD samples, show very mild/no enrichment for transcripts associated with neuronal maintenance-related processes. Oligos1 expresses *RBFOX1*, which encodes an mRNA splicing factor, at high levels. Dysregulation of *RBFOX1* has been implicated in various neurodevelopmental conditions, such as autism, intellectual disability and epilepsy [53]. Upregulation of *RBFOX1*, and dysregulation of splicing, have been reported in PD patient iPSC-derived dopaminergic neurons [54]. The presence of apparently dysfunctional oligodendrocytes in such a high number in PD samples may be PD-specific and contribute to the unique disease pathology.

Certain astrocyte, microglia and oligodendrocyte subpopulations (Astrocytes2, Microglia1 and Oligos2) were observed to be almost absent in PD samples. These glial subpopulations are enriched in *ALDH1A1, SLC6A3, SLC18A2* and *TH*. We were able to confirm that *ALDH1A1*, *SLC6A3* and *TH* are also detected in glial cells using spatial transcriptomics, supporting the existence of *TH* expressing glial populations. In addition, we confirmed the depletion of *TH* + glia populations in PD by performing a meta-analysis on data acquired from an independent sample cohort (Supplementary Figs. 8, 9, 10) [8].

The role of *TH* expression in glial subpopulations is not clear. However, previous studies have suggested that inducing *TH* expression in astrocytes may prevent Parkinsonism in rats [55, 56]. Furthermore, reports have shown that microglia express functional dopamine receptors [57, 58], while expression of DAT, MAO-B, and COMT has been reported in astrocytes, suggesting they can uptake and metabolize dopamine [57]. Furthermore, a study has shown that impaired dopamine homeostasis in the prefrontal cortex, induced by astrocyte-specific knockout of the vesicular monoamine transporter 2 (VMAT2 encoded by *SLC18A2*), adversely impacts synapse formation and function, suggesting that glial regulation of dopaminergic transmission is critical for correct CNS development [59]. Interestingly, Astrocytes2 and Microglia1 express genes involved in apoptosis, oxidative stress and the UPR, while Oligos2 does not. This suggests that the reduction in numbers of Astrocytes2 and Microglia1 in PD may result from the UPR and oxidative stress. Since dopamine oxidation produces reactive quinones and reactive oxygen species that can cause cell damage and death [60], these *TH* enriched PD glia subpopulations might be vulnerable to dopamine-induced oxidative stress. Dopamine has been reported to act as an NLRP3 inflammasome inhibitor in primary human microglia [61]. Reduction of dopamine levels in the brain due to the loss of DA neurons and *TH* enriched glial cells may, therefore, contribute to activated microglia-mediated neuroinflammation and subsequent neurodegeneration.

In summary, our comprehensive snRNA-seq study makes a substantial contribution to our understanding of Parkinson's disease as a complex pathology driven by the interplay of multiple cell types, each displaying distinctive cell type-specific responses. Importantly, key findings from our study of 14 Controls and 15 Sporadic PD donors could not only be validated by spatial transcriptomics on tissue samples from the same cohort but were also consistent with a meta-analysis conducted on the largest snRNA-seq dataset from substantia nigra currently available. Hence, we believe our dataset is a valuable resource for guiding future hypothesis-driven experiments and represents a significant step forward in understanding the cellular dysfunction that drives Parkinson’s disease.

## Methods

### Selection of donors

We selected a total of 29 individuals from the Oregon Brain Bank, a human tissue repository for brain research, from which to obtain tissue for single nucleus sequencing. The Ethics Committee of UZ Leuven/KU Leuven approved the study under the S-number 64182. Fresh frozen tissue was obtained, alongside detailed clinical records. These reports provided information on the donor’s clinical diagnosis, age, sex, as well as details about post-mortem interval (PMI) before the tissue was collected, the quality of RNA (RNA integrity number, RIN), the presence of Lewy bodies in midbrain, limbic (amygdala) and neocortical (frontal cortex) regions, as well as possible AD-related changes in cortex (presence of neuritic plaques and neurofibrillary tangles). This information is summarized in Supplementary Table 1. The 29 samples contained tissue from 14 Control and 15 PD brains. We selected individuals from both sexes (male and female), who were mostly aged 60 + .

### Isolation of nuclei from frozen post-mortem brain tissue and single nucleus RNA-seq

To identify the substantia nigra in Control and PD tissues, cryostat sections of 5 μm thickness were cut, haematoxylin and eosin (H&E) stained and viewed under the microscope by a neuropathologist (D.R.T.) to identify the tissue region containing melanin expressing neurons (substantia nigra pars compacta), which was then manually annotated with a waterproof pen. H&E-guided 2 mm biopsy punches were then collected. Nuclei were isolated using an optimized nucleus isolation protocol [62] and sequencing libraries were prepared using the Chromium Single Cell 3' Reagent Kit v3, according to the manufacturer’s protocol (10X Genomics). The snRNA-seq libraries generated were subsequently sequenced using a NovaSeq6000 system: 1% PhiX, paired-end sequencing with 10X v3 parameters (28-8-0-91 cycles). 13 libraries having a read saturation rate lower than 60% were re-sequenced, using the same 10X parameters, to obtain more reads and improve the quality of the database. Combining reads from both runs gave a > 40% saturation rate for all the samples (Supplementary Table 2).

### Pre-processing single nucleus RNA-seq data

Gene counts were obtained by aligning reads to the human hg38 genome (GRCh38/Ensemble 93 pre-built by 10X Genomics), using CellRanger software (v3.0.2) (10X Genomics). To account for unspliced nuclear transcripts, reads mapping to pre-mRNA were counted (as recommended by CellRanger v.3.0.2). The quantification of pre-mRNA was done using the CellRanger count pipeline on each of the 29 individual libraries, providing double input fastq files for re-sequenced libraries. Default CellRanger parameters were used throughout the pipeline.

### Quality control of single nucleus RNA-seq data

#### Initial processing for downstream identification of major cell types

The initial dataset contained 173,197 nuclei, expressing a total of 30,194 unique transcripts. Thresholds to exclude degrading nuclei with low RNA-content (< 500 transcripts) and doublets having high RNA-content (> 6000 transcripts) were imposed. *MALAT1* was excluded from the transcript list, since it was highly enriched and could bias the clustering. Next, to make sure that only results from single nuclei were left in the database, the Scrublet algorithm [63] was run on each dataset (using default parameters), to further identify nuclei doublets. In total 2,496 doublets were identified (Supplementary Table 2) and removed from the database at this point. Finally, high mitochondrial RNA content was accounted for. A first round of clustering was performed using Seurat v3.1.1 R package [64], limiting the percentage of mitochondrial genes detected per library to 1%, 2% or 5% of total transcripts. This analysis showed a clear bias towards the grouping together of nuclei with high levels of mitochondrial transcripts; this was very apparent in the case of the 5% threshold and became less relevant for the 1% and the 2% thresholds. Hence, to keep the maximum number of nuclei for further analysis, while minimizing the potential for clustering bias, libraries in which 2% or more of the detected transcripts were of putative mitochondrial origin were removed, leaving 83,484 nuclei and 30,194 unique transcripts for downstream analysis.

#### Downstream processing for identification of cell subpopulations

Neuronal and glial populations were subclustered after removing transcripts expressed in less than 10% of nuclei, resulting in the retention of nuclei that expressed between 500 to 6,000 transcripts per nucleus. The percentage of mitochondrial genes detected was re-calculated and libraries containing more than 2% mitochondrial content were removed from further analysis. In the case of neurons, a small subpopulation derived from a single Control individual was removed (188 nuclei from donor s.0147) and the remaining nuclei were re-clustered.

### Single nucleus RNA-seq data—integration of the datasets and clustering

Data normalization and clustering were done with the Seurat v3.1.1 package [64]. First, each dataset was SCT-normalized using the SCTransform() function. The 6000 most variable features were selected for downstream integration using the SelectIntegrationFeatures() function. Principal component analysis (PCA) was performed on each dataset separately with the RunPCA() function, with the top 50 Principal Component (PC) coordinates evaluated. Integration anchors were found using the FindIntegrationAnchors() method, using sample s.0096 as a reference. Datasets were integrated by applying the IntegrateData() function to the top 20 dimensions. PCA was repeated on the integrated database and the top 10 PCs were used for UMAP analysis, using the RunUMAP() function. Finally, the top 10 PCs were used to build a k-nearest-neighbours graph, using the FindNeighbors() function: 0.6 resolution was used to group nuclei in the clusters. Populations enriched for well-known cell type-specific markers were grouped together to define broad cell types. Subclustering of neuronal and glial populations was done based on the top 4 PCs at 0.2 resolution.

### Single nucleus RNA-seq data – cell type enriched marker identification

For each subcluster, marker genes were identified using differential gene expression analysis performed by comparing transcripts expressed by the nuclei within a given subcluster and all the other nuclei defining a given cell population, using a Wilcoxon rank-sum test implementation of Seurat v3.1.1 with a false discovery rate (FDR)-corrected *p*-value < 0.05 and a ln(mean gene expression across cells in a given subcluster/mean gene expression across cells in all other subclusters) of 0.25. Transcripts detected in at least 25% of the nuclei within a given subcluster were considered. The full list of markers is reported in Supplementary Table 5.

### PD gene association analysis

To understand the relationship between cell type-specific gene expression in the substantia nigra and the predisposition to Parkinson’s disease, we obtained a list of high-confidence genes associated with monogenic PD [31]. For analysis of GWAS data, we applied Multi-marker Analysis of GenoMic Annotation (MAGMA) v1.10 on a large Parkinson’s disease GWAS database curated by Nalls and colleagues (using all datasets except those provided by 23andMe) [32]. MAGMA is an enrichment analysis method that tests the joint association of all risk SNPs in a genomic region with a given phenotype, while accounting for the linkage disequilibrium (LD) structure between SNPs [6, 65]. For this study, MAGMA analysis was performed as described by Smajić et al. (2022). We took the SNPs and their *p*-values from the published summary statistics available in Nalls et al. [32] and used the publicly available European subset of the 1000 Genomes project (Phase 3) as a reference to estimate the LD between SNPs. Only the first two steps of the MAGMA workflow were performed. First, SNPs were mapped to genes using the NCBI GRCh37 genome build (annotation release 105). Gene boundaries were defined as the transcribed region of each gene, plus an extended window of 35 kb upstream and 10 kb downstream of each gene to account for potential regulatory elements [66]. Second, we computed the gene-wise *p*-values based on the SNP GWAS *p*-values. The association of a gene with a PD-associated variant is quantified as a *z*-score. *Z*-scores are negative if a gene is not associated with a PD-associated variant, whereas positive association is indicated by a positive *z*-score (MAGMA *p*-value lower than 0.001 is equivalent to a *z*-score value higher than 3). We selected differentially expressed genes for given cell (sub)populations, based on the following criteria: filtered for FDR-corrected *p*-values < 0.05, percentage of cells in a given cluster where expression was detected > 0.1, and ln-fold change > 0.3 (population level) or ln-fold change > 0.25 (subpopulation level). Genes were ordered by average ln-fold change, with a maximum of the top 5 genes showing high association with PD-associated variants shown in the ‘pseudo-Manhattan’ plots in Fig. 6. The full list of genes is reported in Supplementary Table 9.

### Assessing relative cell type enrichment of genes near PD-associated variants

A Binomial test was performed to assess cell type-specific enrichment of genes near PD-associated variants using a two-sided R function ‘binom.test()’, using the parameters reported in Supplementary Table 9. The tests were performed separately for major cell types and glial/neuronal subpopulations, considering markers with the following thresholds: ln-fold change in average expression > 0.3, fraction of the cells expressing the marker in the designated cluster > 0.25, and adjusted *p*-value < 0.05 (Supplementary Table 9).

### Single nucleus RNA-seq data—differential gene-expression analysis among phenotypes

Differential expression analysis, using single nucleus data obtained from PD patients or Control individuals, was performed by fitting a linear mixed model (in which the individual of origin was assigned as a random effect), using the R package lme4. One way ANOVA test with Benjamini–Hochberg correction was performed to assess the statistical significance of the observed up- or downregulation, using the R package Car. The full list of significantly up- or downregulated genes detected by this analysis is reported in Supplementary Table 7.

### Pathway analysis

Over-representation analysis was performed on subpopulation specific marker genes (Figs. 2, 3, 4, 5 panel E; Supplementary Table 6), as well as on the genes differentially expressed between PD and Control conditions (Figs. 2, 3, 4 panel H, Fig. 5 panel G; Supplementary Table 8), using clusterProfiler version 4.4.4 [67]. Over-representation analysis on the common cell marker genes expressed by *TH* + cell types (Fig. 7D) was performed using Metascape [68].

### Spatial transcriptomics—sample preparation

SNpc tissue sections were prepared from the brains of three Control donors and three PD donors, as reported in Supplementary Table 1. Samples were first positioned on the sectioning block and then embedded using FSC 22 frozen section media. A Leica CM3050 cryostat was used to section the samples at a chamber temperature of -18°C and an object temperature of -16°C. The samples were sectioned with a thickness of 10 μm and subsequently placed on pre-chilled coverslips. Following the sectioning process, the samples were stored at -80°C and then shipped to Resolve GmbH on dry ice for additional processing.

### Spatial transcriptomics—molecular cartography

Spatial transcriptomics was performed using Resolve Biosciences’ commercially available Molecular Cartography™ platform. Sections were hybridized with probes specifically targeting 96 unique transcripts (Supplementary Table 4). Following hybridization, samples were washed to remove excess probes and then fluorescently labelled through a two-step color development process. Regions were imaged and the fluorescent signal was removed during the decolorization step. This cycle of color development, imaging and decolorization was repeated to generate a unique combinatorial code for each target transcript. Images with DAPI staining, alongside the corresponding coordinates of each transcript on the image, were provided. The coordinate files served as input for segmentation using Baysor [69].

### Spatial transcriptomics—object segmentation

Segmentation was performed in two steps using Baysor version 0.5.2. First, the molecular data (x, y, z coordinates) was segmented with the following parameters: cell radius (45 pixels), cell radius standard deviation (25%) and a minimum number of molecules per cell (15). However, it is known that Baysor may over-segment objects that have large areas, often resulting in multiple smaller objects being identified instead of a single large object. To ensure proper segmentation, we then repeated the process using a radius of 90 pixels. In this step, only molecules considered as noise (parameter given as output by Baysor) during the initial segmentation and objects having an area larger than 80% of the calculated area (0.8 * π * 45px^2^) were used as input. Images were classed as correctly segmented when the number of output objects was smaller than the number of large objects used as input. If not, the large objects from the initial segmentation were considered as properly segmented. Properly segmented images were then used to generate information on the transcript counts per cell, as well as single molecule and cell meta-data.

### Spatial transcriptomics—quality control

A total of 75,416 cells remained after removing objects with undefined area or infinite density. Objects were retained if they contained more than 10 molecules, originating from at least 2 unique genes, had a molecule-to-gene ratio higher than 1, elongation below 10, density below 0.04, average confidence above 0.7 and area larger than 500 pixels. These thresholds were chosen based on visual examination of each variable’s numeric distribution, plotted using histograms. No maximum area threshold was defined, due to some cells having large sizes. This resulted in 41,198 segmented objects. An initial clustering quality check was performed by clustering the filtered objects using Seurat v4.3.0 with the SCT algorithm, followed by Harmony batch effect correction. Parameters were set as follows: scaleFactor (10,000), nFeatures (200), PCA components (13), resolution (0.3), variable features (3,000) and residualVariance cutoff (1.3). Variables corrected by Harmony included batch variations (batchID: batch 1 and batch 2), differences between imaged sections (tissueSection_ID) and selected regions of interest (tissueROI_ID).

### Spatial transcriptomics—cell type classification and clustering

Cell type identification was performed using a unimodal UMAP projection workflow run in Seurat v4.3.0, which allows projection of a query dataset (spatial) onto a reference UMAP structure (snRNA-seq dataset described in this manuscript). The reference dataset was downsampled to 26,907 nuclei - sampling 5,000 nuclei from each cell type, except for vascular cells (1,560) and T-cells (347). Anchors between the query and reference datasets were determined using the FindTransferAnchors function, using CCA reduction, SCT normalization and 40 PCA dimensions. These anchors were imported into the MapQuery function with cellType_L1 as a class variable, again using CCA reduction and the UMAP reduction model. Following mapping, a secondary filtering step was performed: within each UMAP-defined spatial cluster, outlier cells that did not belong to the predominant population were identified and removed. For example, in a cluster composed by microglia (green dots), a few interspersed oligodendrocytes (blue dots) were removed to ensure the homogeneity of the cluster assignment. This filtering step resulted in 36,806 high-quality cells. Final clustering and batch effects correction were performed as detailed in the ‘Spatial transcriptomics - quality control’ section.

### Meta-data analysis

To compare the similarity between the glia subpopulations identified in our study and those populations found in published datasets, we performed a comprehensive meta-analysis, focusing on the snRNA-seq data published by Kamath et al. [8]. We extracted and processed the gene expression data for the major glial cell types: microglia, astrocytes and oligodendrocytes. To identify differentially expressed genes for each subpopulation, we used the FindAllMarkers function in Seurat v4.3.0, using default parameters. For individual subpopulations in this dataset, for instance microglia MG_CECR2_FLG11, we retained only genes that met the following criterion: significant enrichment (adjusted *p*-value < 0.05) in any subpopulation of the same major cell type (Microglia0, Microglia1, Microglia2, Microglia3, Microglia4 and Microglia5) in our study (Supplementary Table 5). To evaluate the expression profiles of each gene set, we used the AddModuleScore function from Seurat v4.3.0. This function provides a measure of enrichment for a defined set of genes within a group of individual nuclei. We plot the percentage of nuclei from each subpopulation with a module score of 0.5 or higher in Supplementary Figs. 8, 9, 10, panel E and show the module scores as feature plots in Supplementary Figs. 8, 9, 10, panel F.

### Supplementary Information

**Additional file 1:** **Supplementary Table 1.** Description of donor material and tissue usage.

**Additional file 2:** **Supplementary Table 2.** Single nucleus RNA-seq (snRNA-seq) quality assessment and the distribution of recovered nuclei across cell types.

**Additional file 3:** **Supplementary Table 3.** Cell proportion analysis (major cell types and subpopulations of neurons, astrocytes, microglia and oligodendrocytes).

**Additional file 4:** **Supplementary Table 4.** List of spatial transcriptomics probes used in this study.

**Additional file 5:** **Supplementary Table 5.** Differentially expressed genes in higher order cell types identified based on snRNA-seq (SN) or spatial transcriptomics (ST) data. Differentially expressed genes in neuronal, astrocyte, microglial and oligodendrocyte subpopulations identified based on the snRNA-seq data.

**Additional file 6:** **Supplementary Table 6.** Pathway (GO and KEGG) over-representation analysis on the cell type markers reported in Supplementary Table 5.

**Additional file 7:** **Supplementary Table 7.** Differentially expressed genes in neuronal, astrocyte, microglial and oligodendrocyte subpopulations when comparing PD to Control samples.

**Additional file 8:** **Supplementary Table 8.** Pathway (GO and KEGG) over-representation analysis on the differentially expressed genes reported in Supplementary Table 7.

**Additional file 9:** **Supplementary Table 9.** A list of high-confidence monogenic PD genes, as reported by Blauwendraat et al. (2020), which was used in this study. Also listed is the MAGMA gene analysis output generated using the Nalls et al. (2019) SNP data, as well as the results of a Binomial test conducted to assess enrichment of genes near PD-associated variants in various cell types.

**Additional file 10:** **Supplementary Table 10.** Pathway (GO and KEGG) over-representation analysis on the genes reported in Supplementary Table 5, which are commonly expressed across the *TH *enriched subpopulations depleted in PD (Neurons0, Astrocytes2, Microglia1, and Oligos2).

**Additional file 11:** **Supplementary Figure 1.** High level cell type clustering. (A) The expression pattern of key markers that identify higher order cell types. SCT-normalized expression levels are shown except for the nFeatures, which refers to the number of genes reporting non-zero expression in a given nucleus. (B) Proportion of nuclei coming from Control (red) and PD (blue) patients for each cell type reported in Figure 1B. Different shades of blue and red represent different donors.

**Additional file 12:** **Supplementary Figure 2.** Landscape of single-nucleus RNA datasets for human midbrain. (A) Summary table of main parameters. SN, substantia nigra; SNpc, substantia nigra pars compacta; MB, midbrain; NA, information not available. (B) Total number of nuclei sequenced and assigned to various cell populations. DA, dopaminergic neurons; non-DA, non-dopaminergic neurons. Only nuclei from Control and PD donors were included in the assessment. Data obtained from nuclei extracted from patients diagnosed with Lewy body dementia were excluded. (C) Number of *TH*+ nuclei across cell populations.

**Additional file 13:** **Supplementary Figure 3.** Markers for neuronal subpopulations grouped by enriched terms reported in Supplementary Table 6, as well as *AGTR1, *which is reported by Kamath et al. [8] to be a marker for a dopaminergic neuron subpopulation vulnerable to degeneration in PD.

**Additional file 14:** **Supplementary Figure 4.** Markers for astrocyte subpopulations grouped by enriched terms reported in Supplementary Table 6.

**Additional file 15:** **Supplementary Figure 5.** Markers for microglial subpopulations grouped by enriched terms reported in Supplementary Table 6.

**Additional file 16:** **Supplementary Figure 6.** Markers for oligodendrocyte subpopulations grouped by enriched terms reported in Supplementary Table 6.

**Additional file 17:** **Supplementary Figure 7.** PD-associated genes and genes near PD-associated variants show cell type-specific expression patterns. (A) Subpopulation specific enrichment of high-confidence genes associated with monogenic PD. Ln-fold change of SCT-normalized counts is shown (Wilcoxon test, *FDR-corrected *p*-value < 0.05; see Supplementary Table 5). (B) Confirmation of cell type-specific enrichment of PD-associated genes using spatial transcriptomics; Log_2_-fold change of SCT-normalized counts is shown. A full list of markers of higher order cell types identified through spatial transcriptomics is provided in Supplementary Table 5. (C) Population specific enrichment of selected genes near PD-associated variants identified by MAGMA analysis on GWAS data. Ln-fold changes in SCT-normalized counts are shown. (Wilcoxon test, *FDR-corrected *p*-value < 0.05, see Supplementary Table 5).

**Additional file 18:** **Supplementary Figure 8.** Meta-analysis of astrocyte subpopulations. (A) UMAP of astrocyte subpopulations identified in our study. (B) UMAP of astrocyte subpopulations identified in the Kamath et al. study. (C) Bar plot showing the total number of *TH*-positive (*TH*+) astrocytes isolated from healthy Controls (CTR) or Parkinson’s disease (PD) samples in our study (left) and the number isolated from Controls (Ctrl), Lewy body dementia (LBD) samples and PD samples in Kamath et al. (right). (D) Bar plot showing the number of *TH*-positive (*TH*+) astrocytes at the subpopulation level in our dataset (left) and the Kamath et al. dataset (right). (E) Heatmap showing the percentage of individual cells in each subpopulation identified in our study (x-axis) that shows an enrichment score of 0.5 or higher. The enrichment score measures the enrichment of subpopulation gene sets extracted from Kamath et al. (y-axis) (see ‘Methods’). (F) Feature plots showing the distribution of the module scores using the UMAP space shown in (A).

**Additional file 19:** **Supplementary Figure 9.** Meta-analysis of microglia subpopulations. (A) UMAP of microglia subpopulations identified in our study. (B) UMAP of microglia subpopulations identified in the Kamath et al. study. (C) Bar plot showing the total number of *TH*-positive (*TH*+) microglia isolated from healthy Controls (CTR) or Parkinson’s disease (PD) samples in our study (left) and the number isolated from Controls (Ctrl), Lewy body dementia (LBD) samples and PD samples in Kamath et al. (right). (D) Bar plot showing the number of *TH*-positive (*TH*+) microglia at the subpopulation level in our dataset (left) and the Kamath et al. dataset (right). (E) Heatmap showing the percentage of individual cells in each subpopulation identified in our study (x-axis) that shows an enrichment score of 0.5 or higher. The enrichment score measures the enrichment of subpopulation gene sets extracted from Kamath et al. (y-axis) (see ‘Methods’). (F) Feature plots showing the distribution of the module scores using the UMAP space shown in (A).

**Additional file 20:** **Supplementary Figure 10.** Meta-analysis of oligodendrocyte subpopulations. (A) UMAP of oligodendrocyte subpopulations identified in our study. (B) UMAP of oligodendrocyte subpopulations identified in the Kamath et al. study. (C) Bar plot showing the total number of *TH*-positive (*TH*+) oligodendrocytes isolated from healthy Controls (CTR) or Parkinson’s disease (PD) samples in our study (left) and the number isolated from Controls (Ctrl), Lewy body dementia (LBD) samples and PD samples in Kamath et al. (right). (D) Bar plot showing the number of *TH*-positive (*TH*+) oligodendrocytes at the subpopulation level in our dataset (left) and the Kamath et al. dataset (right). (E) Heatmap showing the percentage of individual cells in each subpopulation identified in our study (x-axis) that shows an enrichment score of 0.5 or higher. The enrichment score measures the enrichment of subpopulation gene sets extracted from Kamath et al. (y-axis) (see ‘Methods’). (F) Feature plots showing the distribution of the module scores using the UMAP space shown in (A).

**Additional file 21:** **Supplementary Figure 11.** *TH* expression across subpopulations. Violin plots showing the SCT-normalized expression of tyrosine hydroxylase (*TH*) across subpopulations of neurons, astrocytes, microglia and oligodendrocytes.

## Data Availability

snRNA-seq data files can be accessed at https://www.ncbi.nlm.nih.gov/geo/query/acc.cgi?acc=GSE243639. Spatial transcriptomics data files are available at 10.5281/zenodo.10451502.

## Abbreviations

AD: Alzheimer’s disease
ALS: Amyotrophic lateral sclerosis
Astro: Astrocytes
CAMK2G: Calmodulin-dependent protein kinase II gamma
CHST1: Carbohydrate sulfotransferase 1
CNS: Central nervous system
COG4: Component of Oligomeric Golgi Complex 4
DA: Dopaminergic
DEG: Differentially expressed gene
ER: Endoplasmic reticulum
FDR: False discovery rate
GWAS: Genome-wide association study
H&E: Haematoxylin and eosin
HLA: Human leukocyte antigen
HPRT1: Hypoxanthine guanine phosphoribosyltransferase
LBD: Lewy body dementia
LD: Linkage disequilibrium
MAGMA: Multi-marker analysis of GenoMic annotation
Micro: Microglia
OGA: O-GlcNAcase
Oligo: Oligodendrocytes
OPC: Oligodendrocyte progenitor cells
PC: Principal component
PCA: Principal component analysis
PD: Parkinson’s disease
PEG10: Paternally expressed gene 10
PMI: Post-mortem interval
RIN: RNA integrity number
RNA-seq: RNA sequencing
sHSP: Small heat shock protein
SN: Substantia nigra
SNpc: Substantia nigra pars compacta
SNPs: Single nucleotide polymorphisms
snRNA-seq: Single nucleus RNA-sequencing
ST: Spatial transcriptomics
TH: Tyrosine hydroxylase
TNF: Tumor necrosis factor
TNKS2: TRF1-interacting ankyrin-related ADP-ribose polymerase 2
TOMM40: Translocase of outer mitochondrial membrane 40
UPR: Unfolded protein response
VC: Vascular cells
VMAT2: Vesicular monoamine transporter 2
