# Correction: Unravelling cell type-specific responses to Parkinson’s Disease at single cell resolution

**DOI:** 10.1186/s13024-024-00717-9

**Published:** 2024-03-25

**Authors:** Araks Martirosyan, Rizwan Ansari, Francisco Pestana, Katja Hebestreit, Hayk Gasparyan, Razmik Aleksanyan, Silvia Hnatova, Suresh Poovathingal, Catherine Marneffe, Dietmar R. Thal, Andrew Kottick, Victor J. Hanson-Smith, Sebastian Guelfi, William Plumbly, T. Grant Belgard, Emmanouil Metzakopian, Matthew G. Holt

**Affiliations:** 1 VIB Center for Brain & Disease Research, KU Leuven, Leuven, Belgium; 2 Department of Clinical Neurosciences, UK Dementia Research Institute, University of Cambridge, CB2 0AH Cambridge, UK; 3 Verge Genomics, South San Francisco, CA USA; 4 Armenian Bioinformatics Institute, Yerevan, Armenia; 5 Department of Mathematics and Mechanics, Yerevan State University, Yerevan, Armenia; 6 Laboratory for Neuropathology, Department of Imaging and Pathology, Leuven Brain Institute, Department of Pathology, KU Leuven, UZ Leuven, Leuven, Belgium; 7 The Bioinformatics CRO, Orlando, FL USA; 8 bit.bio, The Dorothy Hodgkin Building, Babraham Research Institute, Cambridge CB223FH,, Cambridge, UK; 9 Laboratory of Synapse Biology, i3S, Porto, Portugal

Molecular Neurodegeneration (2024) 19:7

https://doi.org/10.1186/s13024-023-00699-0.

The original article contained an error whereby the production team handling the article mistakenly omitted the company of affiliation #8 (‘ bit.bio’).

The affiliation text has since been corrected, and is also viewable in this Correction article.

