# Supplementary figures and images for: Unravelling cell type-specific responses to Parkinson’s Disease at single cell resolution
Source: Mol Neurodegener. 2024 Jan 20;19:7. doi: 10.1186/s13024-023-00699-0 (PMC10799528; doi:10.1186/s13024-023-00699-0)

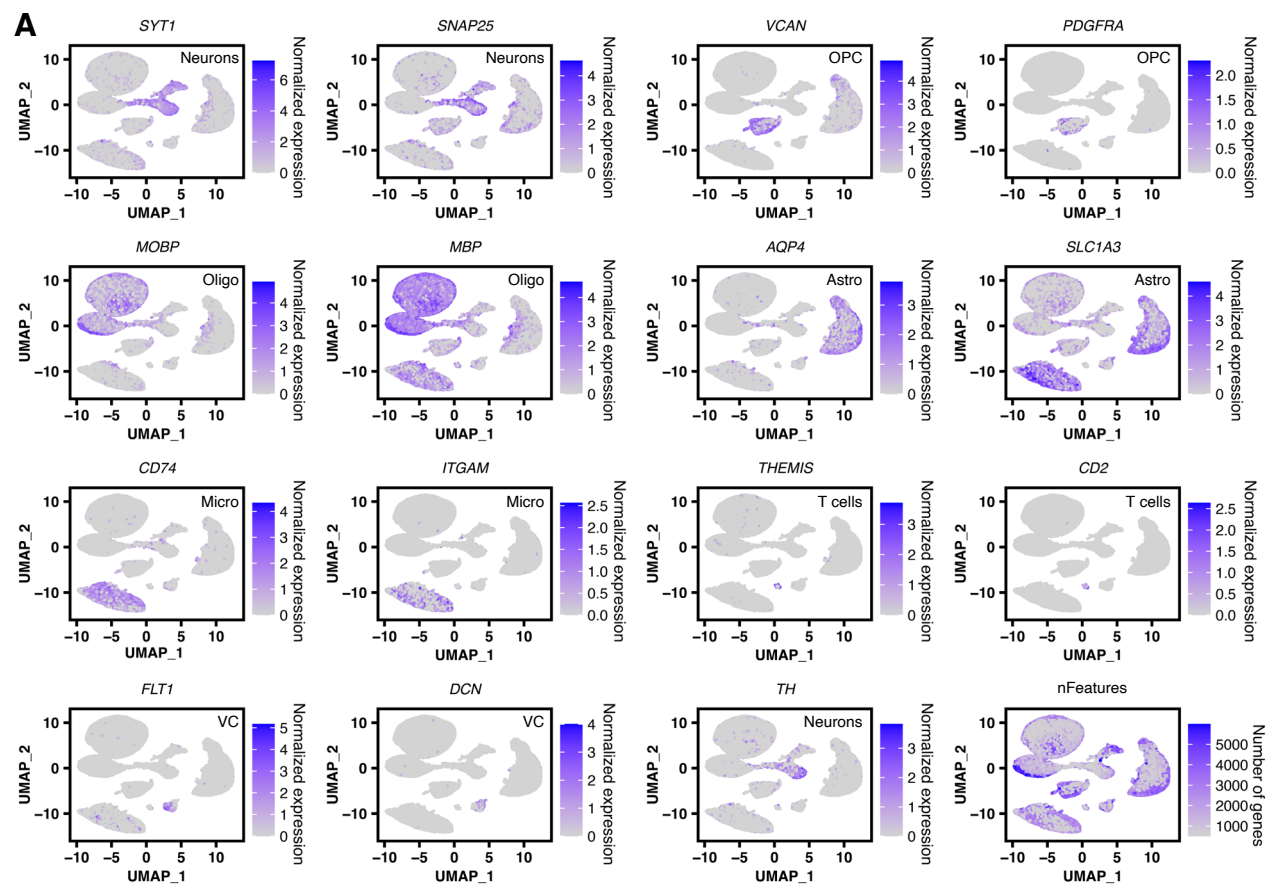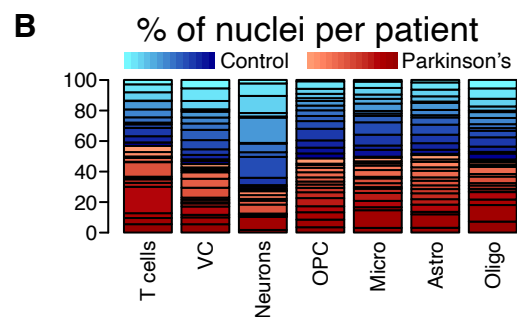

Supplement: Supplementary file 11 — Additional file 11: Supplementary Figure 1. High level cell type clustering. (A) The expression pattern of key markers that identify higher order cell types. SCT-normalized expression levels are shown except for the nFeatures, which refers to the number of genes reporting non-zero expression in a given nucleus. (B) Proportion of nuclei coming from Control (red) and PD (blue) patients for each cell type reported in Figure 1B. Different shades of blue and red represent different donors. [file 13024_2023_699_MOESM11_ESM.pdf]

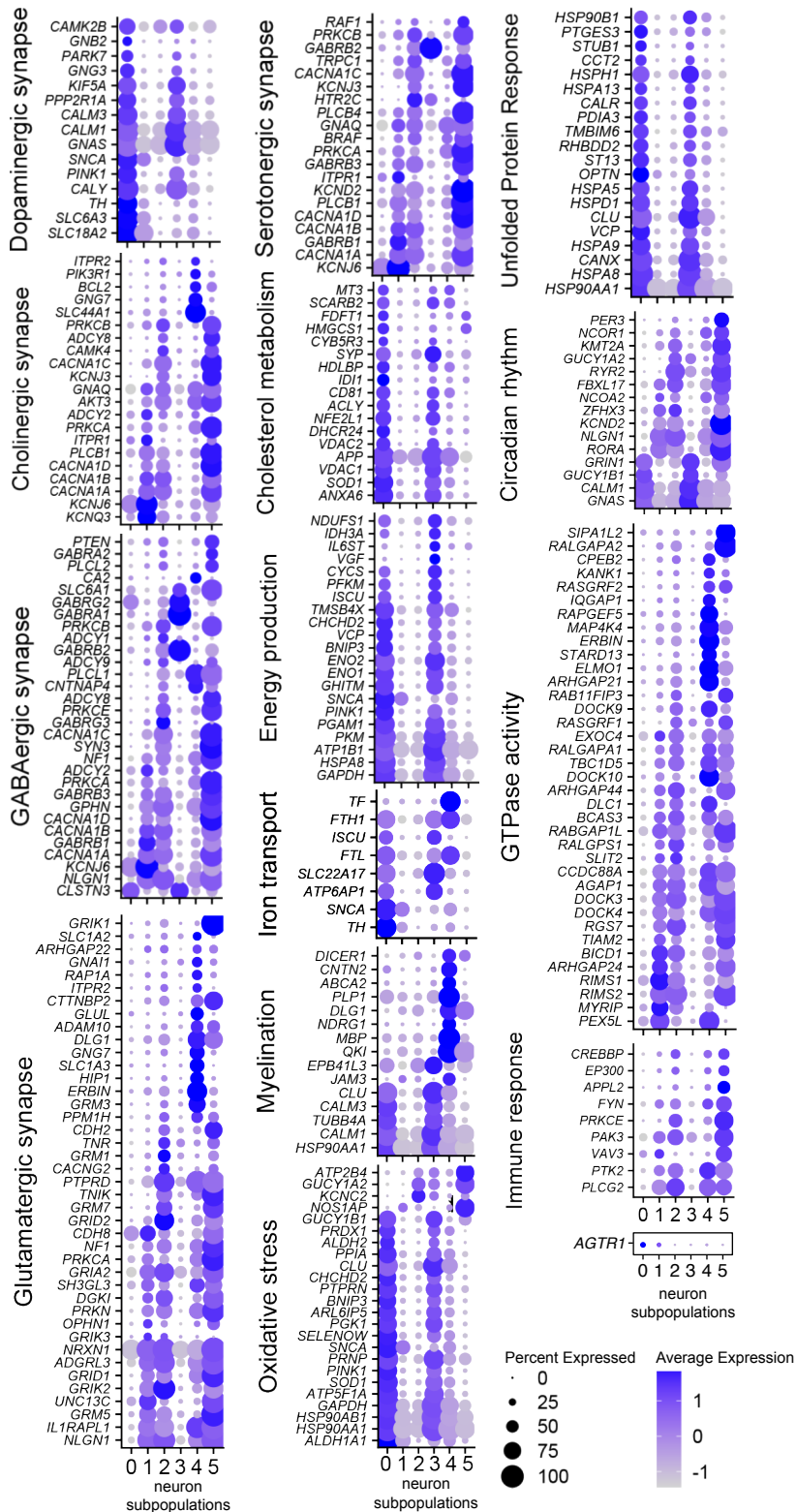

Supplementary Figure 3\_Martirosyan, Ansari & Pestana et al., 2023

Supplement: Supplementary file 13 — Additional file 13: Supplementary Figure 3. Markers for neuronal subpopulations grouped by enriched terms reported in Supplementary Table 6, as well as AGTR1, which is reported by Kamath et al. [8] to be a marker for a dopaminergic neuron subpopulation vulnerable to degeneration in PD. [file 13024_2023_699_MOESM13_ESM.pdf]

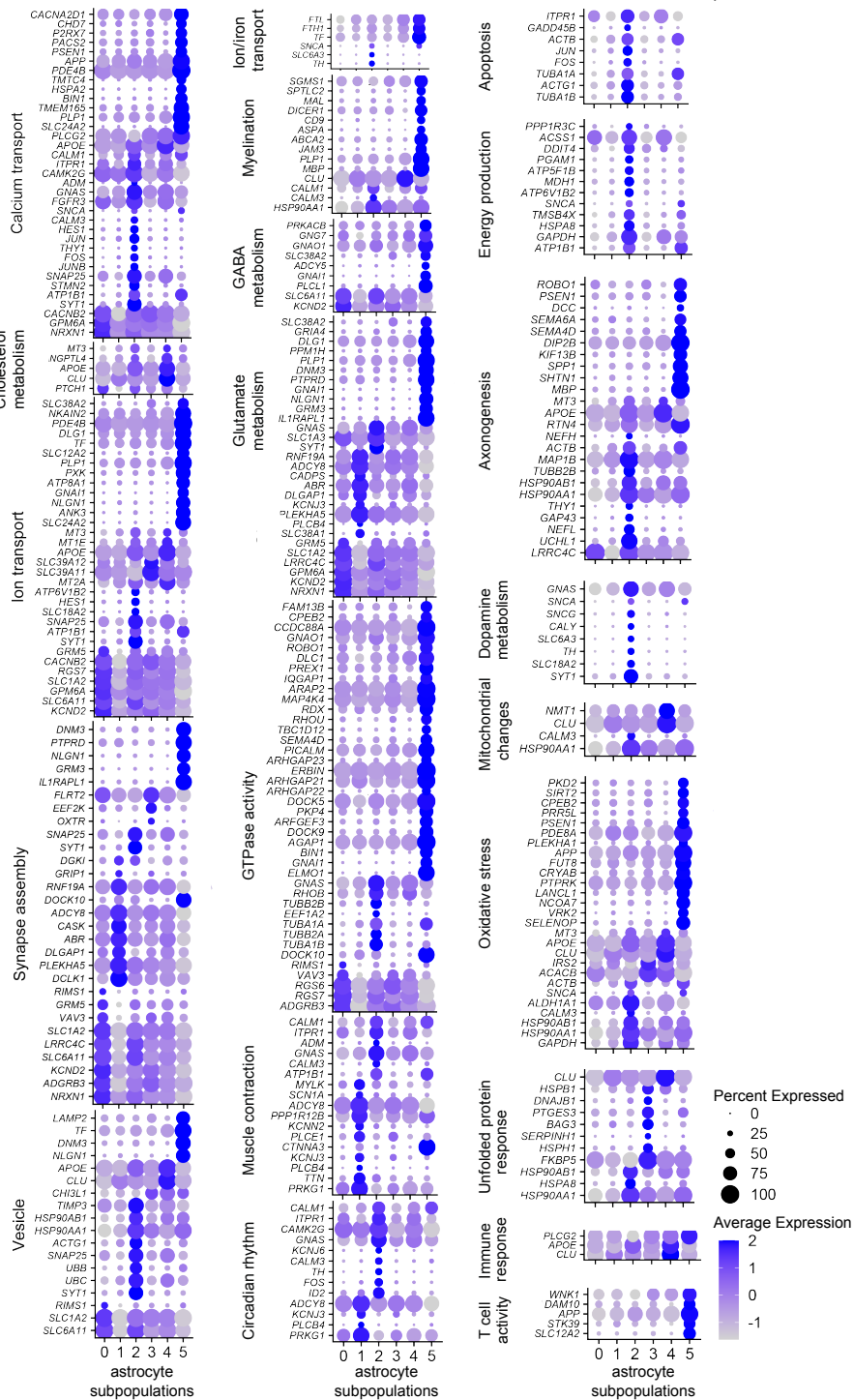

Supplement: Supplementary file 14 — Additional file 14: Supplementary Figure 4. Markers for astrocyte subpopulations grouped by enriched terms reported in Supplementary Table 6. [file 13024_2023_699_MOESM14_ESM.pdf]

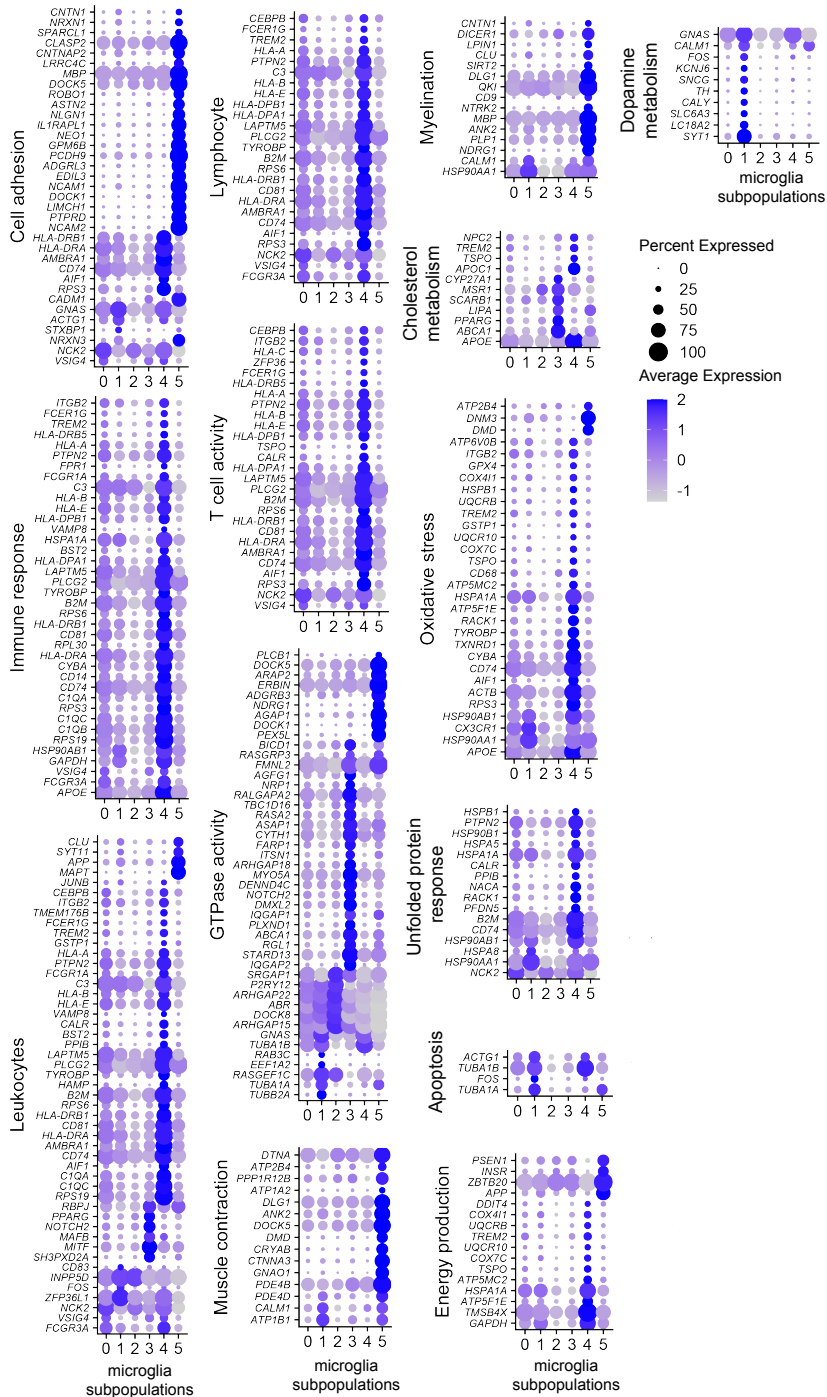

Supplementary Figure 5\_Martirosyan, Ansari & Pestana et al., 2023

Supplement: Supplementary file 15 — Additional file 15: Supplementary Figure 5. Markers for microglial subpopulations grouped by enriched terms reported in Supplementary Table 6. [file 13024_2023_699_MOESM15_ESM.pdf]

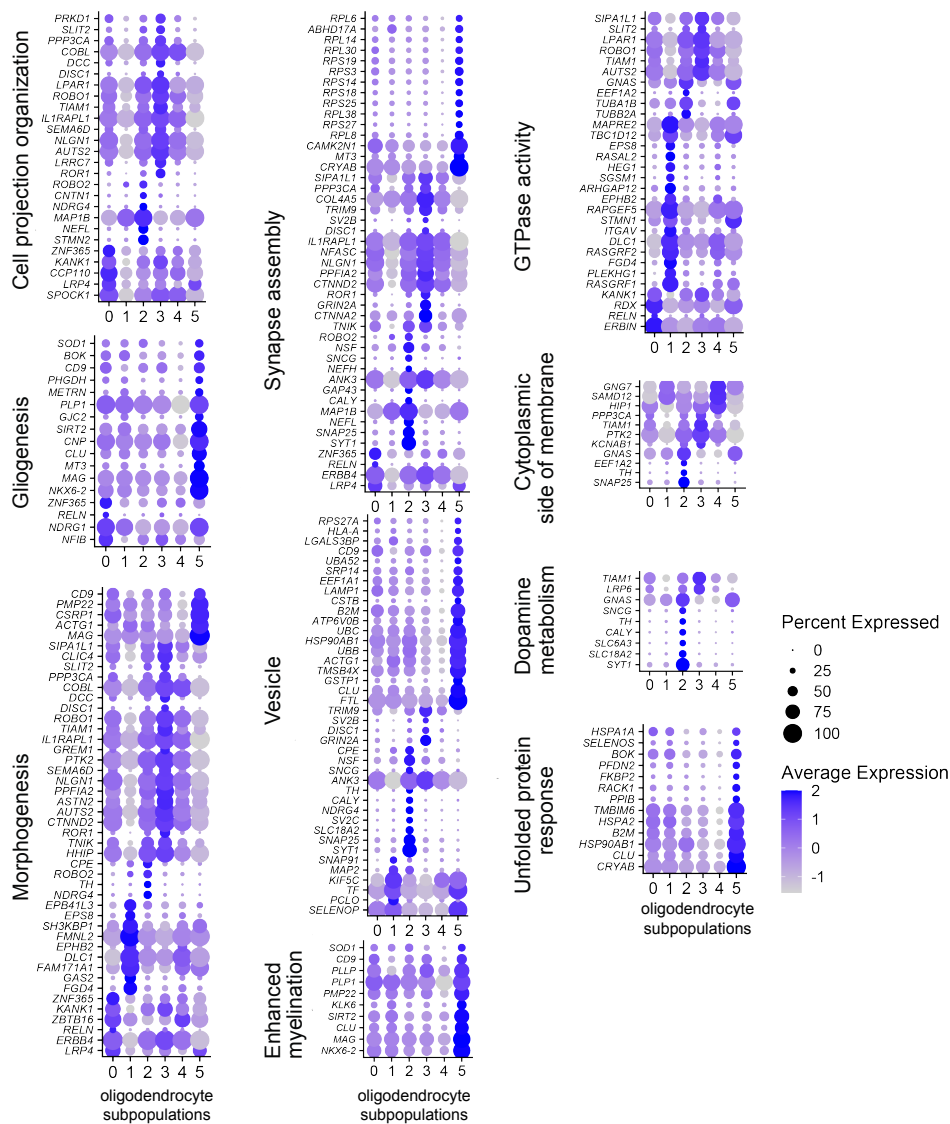

Supplement: Supplementary file 16 — Additional file 16: Supplementary Figure 6. Markers for oligodendrocyte subpopulations grouped by enriched terms reported in Supplementary Table 6. [file 13024_2023_699_MOESM16_ESM.pdf]

## A Monogenic genes

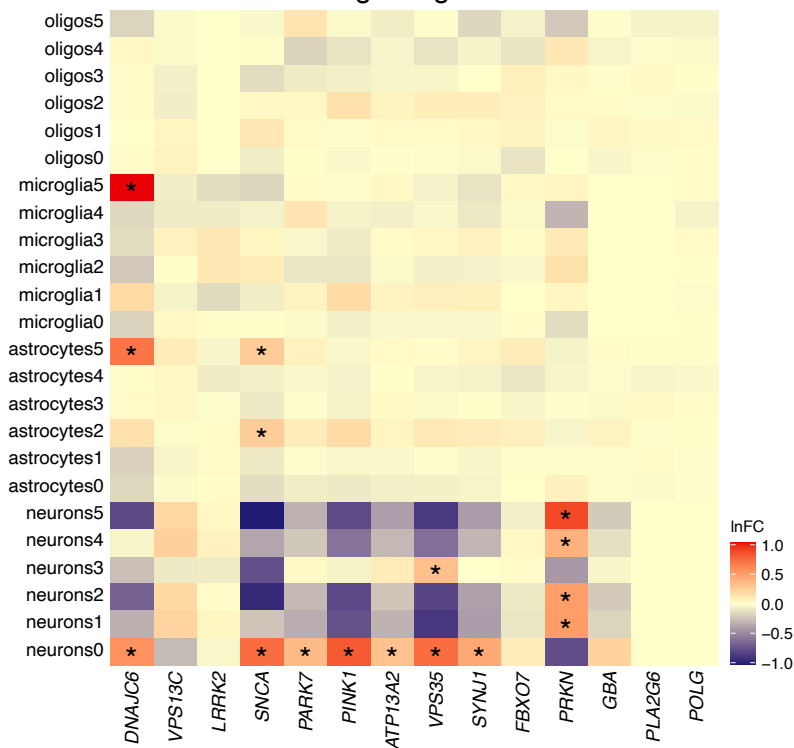

## B Spatial transcriptomics

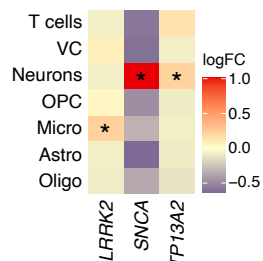

## C GWAS

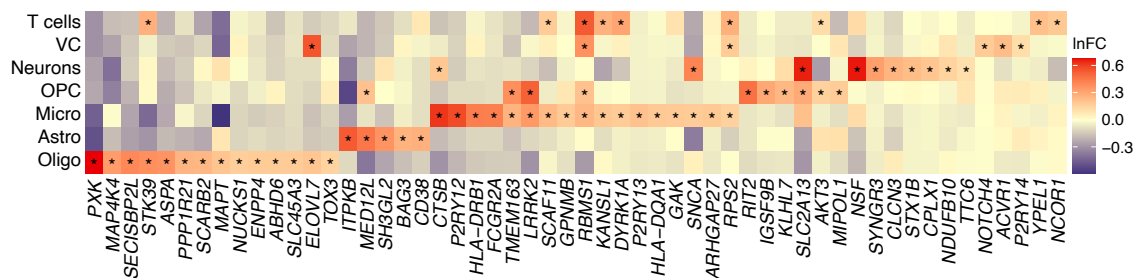

Supplement: Supplementary file 17 — Additional file 17: Supplementary Figure 7. PD-associated genes and genes near PD-associated variants show cell type-specific expression patterns. (A) Subpopulation specific enrichment of high-confidence genes associated with monogenic PD. Ln-fold change of SCT-normalized counts is shown (Wilcoxon test, *FDR-corrected p-value < 0.05; see Supplementary Table 5). (B) Confirmation of cell type-specific enrichment of PD-associated genes using spatial transcriptomics; Log2-fold change of SCT-normalized counts is shown. A full list of markers of higher order cell types identified through spatial transcriptomics is provided in Supplementary Table 5. (C) Population specific enrichment of selected genes near PD-associated variants identified by MAGMA analysis on GWAS data. Ln-fold changes in SCT-normalized counts are shown. (Wilcoxon test, *FDR-corrected p-value < 0.05, see Supplementary Table 5). [file 13024_2023_699_MOESM17_ESM.pdf]

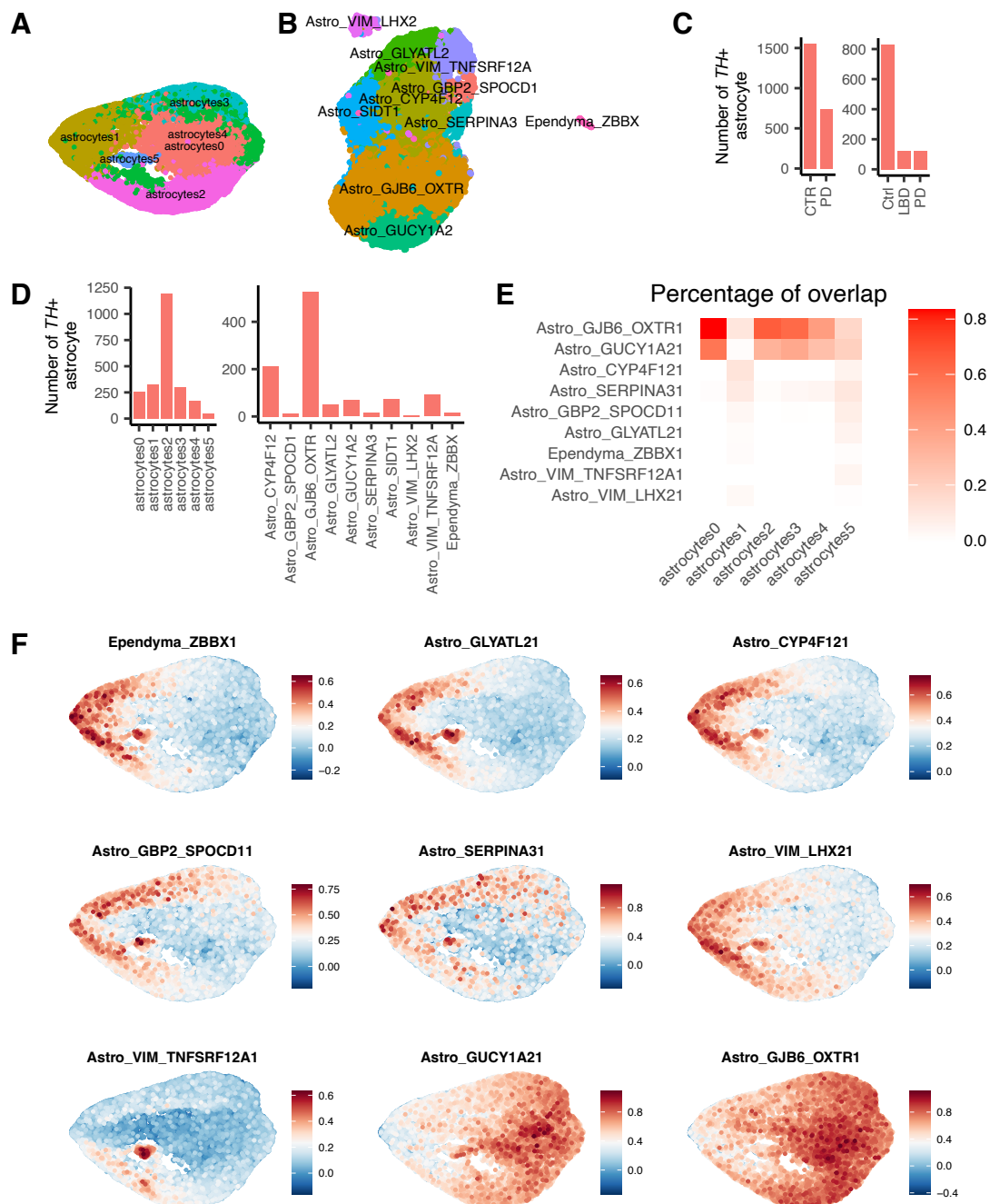

Supplementary Figure 8\_Martirosyan, Ansari & Pestana et al., 2023

Supplement: Supplementary file 18 — Additional file 18: Supplementary Figure 8. Meta-analysis of astrocyte subpopulations. (A) UMAP of astrocyte subpopulations identified in our study. (B) UMAP of astrocyte subpopulations identified in the Kamath et al. study. (C) Bar plot showing the total number of TH-positive (TH+) astrocytes isolated from healthy Controls (CTR) or Parkinson’s disease (PD) samples in our study (left) and the number isolated from Controls (Ctrl), Lewy body dementia (LBD) samples and PD samples in Kamath et al. (right). (D) Bar plot showing the number of TH-positive (TH+) astrocytes at the subpopulation level in our dataset (left) and the Kamath et al. dataset (right). (E) Heatmap showing the percentage of individual cells in each subpopulation identified in our study (x-axis) that shows an enrichment score of 0.5 or higher. The enrichment score measures the enrichment of subpopulation gene sets extracted from Kamath et al. (y-axis) (see ‘Methods’). (F) Feature plots showing the distribution of the module scores using the UMAP space shown in (A). [file 13024_2023_699_MOESM18_ESM.pdf]

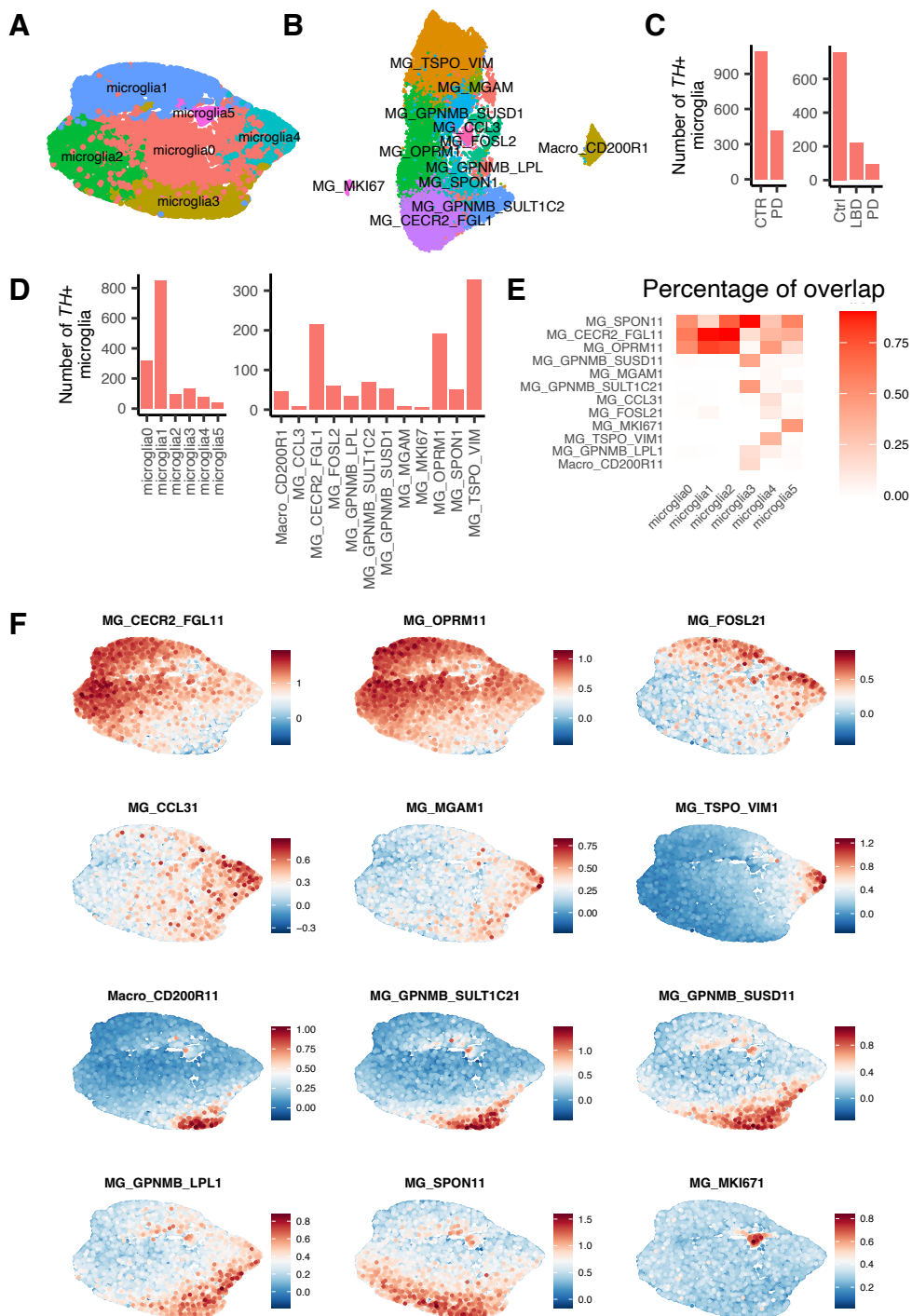

Supplement: Supplementary file 19 — Additional file 19: Supplementary Figure 9. Meta-analysis of microglia subpopulations. (A) UMAP of microglia subpopulations identified in our study. (B) UMAP of microglia subpopulations identified in the Kamath et al. study. (C) Bar plot showing the total number of TH-positive (TH+) microglia isolated from healthy Controls (CTR) or Parkinson’s disease (PD) samples in our study (left) and the number isolated from Controls (Ctrl), Lewy body dementia (LBD) samples and PD samples in Kamath et al. (right). (D) Bar plot showing the number of TH-positive (TH+) microglia at the subpopulation level in our dataset (left) and the Kamath et al. dataset (right). (E) Heatmap showing the percentage of individual cells in each subpopulation identified in our study (x-axis) that shows an enrichment score of 0.5 or higher. The enrichment score measures the enrichment of subpopulation gene sets extracted from Kamath et al. (y-axis) (see ‘Methods’). (F) Feature plots showing the distribution of the module scores using the UMAP space shown in (A). [file 13024_2023_699_MOESM19_ESM.pdf]

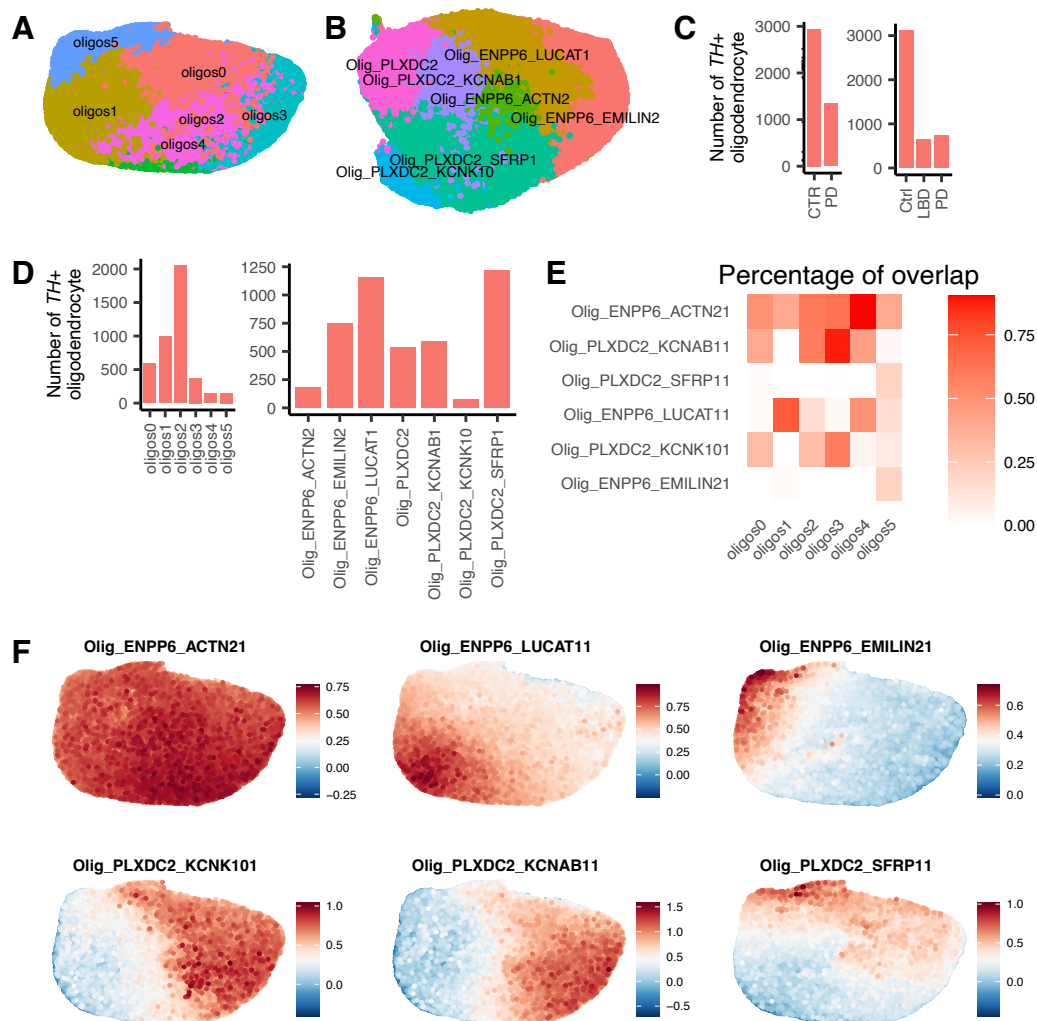

Supplement: Supplementary file 20 — Additional file 20: Supplementary Figure 10. Meta-analysis of oligodendrocyte subpopulations. (A) UMAP of oligodendrocyte subpopulations identified in our study. (B) UMAP of oligodendrocyte subpopulations identified in the Kamath et al. study. (C) Bar plot showing the total number of TH-positive (TH+) oligodendrocytes isolated from healthy Controls (CTR) or Parkinson’s disease (PD) samples in our study (left) and the number isolated from Controls (Ctrl), Lewy body dementia (LBD) samples and PD samples in Kamath et al. (right). (D) Bar plot showing the number of TH-positive (TH+) oligodendrocytes at the subpopulation level in our dataset (left) and the Kamath et al. dataset (right). (E) Heatmap showing the percentage of individual cells in each subpopulation identified in our study (x-axis) that shows an enrichment score of 0.5 or higher. The enrichment score measures the enrichment of subpopulation gene sets extracted from Kamath et al. (y-axis) (see ‘Methods’). (F) Feature plots showing the distribution of the module scores using the UMAP space shown in (A). [file 13024_2023_699_MOESM20_ESM.pdf]

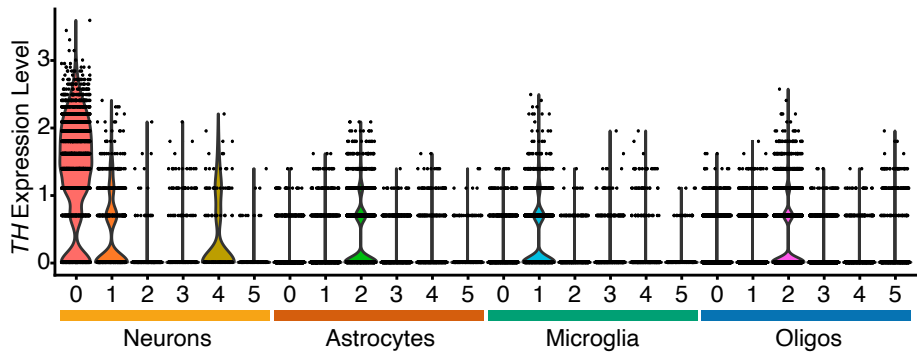

Supplement: Supplementary file 21 — Additional file 21: Supplementary Figure 11. TH expression across subpopulations. Violin plots showing the SCT-normalized expression of tyrosine hydroxylase (TH) across subpopulations of neurons, astrocytes, microglia and oligodendrocytes. [file 13024_2023_699_MOESM21_ESM.pdf]
