# Supplementary material for: Unravelling cell type-specific responses to Parkinson’s Disease at single cell resolution
Source: Mol Neurodegener. 2024 Jan 20;19:7. doi: 10.1186/s13024-023-00699-0 (PMC10799528; doi:10.1186/s13024-023-00699-0)
Supplement: Supplementary file 12 — Additional file 12: Supplementary Figure 2. Landscape of single-nucleus RNA datasets for human midbrain. (A) Summary table of main parameters. SN, substantia nigra; SNpc, substantia nigra pars compacta; MB, midbrain; NA, information not available. (B) Total number of nuclei sequenced and assigned to various cell populations. DA, dopaminergic neurons; non-DA, non-dopaminergic neurons. Only nuclei from Control and PD donors were included in the assessment. Data obtained from nuclei extracted from patients diagnosed with Lewy body dementia were excluded. (C) Number of TH+ nuclei across cell populations. [file 13024_2023_699_MOESM12_ESM.pdf]

A

	Agarwal et al., 2020	Smajic et al., 2022	Kamath et al., 2022	Our study
Number of nuclei	5,943	41,435	320,016	83,484
Number of donors	5 CTR	6 CTR/5 PD	8 CTR/6 PD	14 CTR/15 PD
Brain region	SN	MB	SN	SNpc
Version	10X v2	10X v3.1	10X v3	10X v3
Unique transcripts detected	26,565	26,725	37,389	30,194
Sequencing depth	43,150	NA	NA	41,360

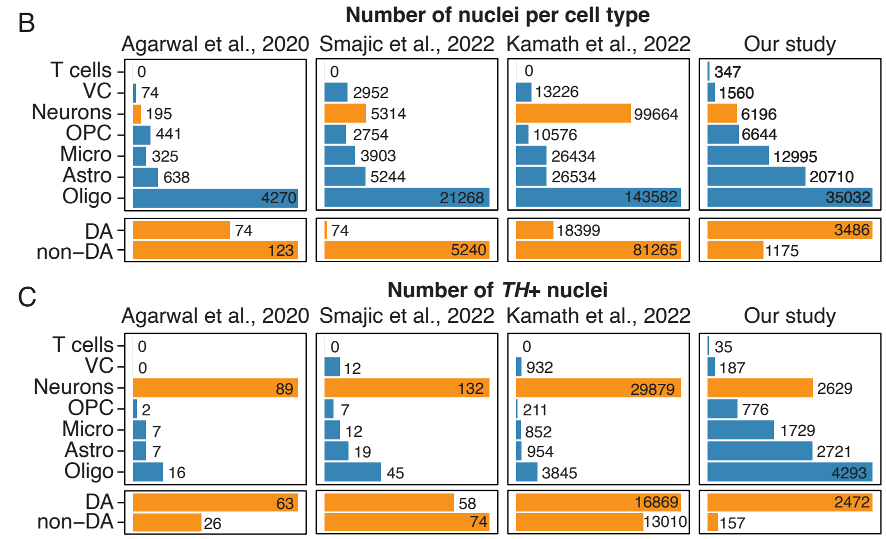

Supplementary Figure 2_Martirosyan, Ansari & Pestana et al., 2023
